# PIWIs Regulate Spermatogonia Self-Renewal and Differentiation by Wnt/β-Catenin Signaling Pathway in *Eriocheir sinensis*

**DOI:** 10.3390/biology14101440

**Published:** 2025-10-18

**Authors:** Bang-Hong Wei, Zhan Zhao, Hong-Yu Qi, Zhen-Fang Li, Wan-Xi Yang, Shuang-Li Hao

**Affiliations:** 1 College of Marine Sciences, South China Agricultural University, Guangzhou 510642, China; 2 The Sperm Laboratory, College of Life Sciences, Zhejiang University, Hangzhou 310058, China; weibanghong@spermlab.org (B.-H.W.);

**Keywords:** *Eriocheir sinensis*, PIWI, Wnt-signaling pathway, proliferation, spermatogonium

## Abstract

**Simple Summary:**

PIWI (P-element-induced wimpy testis) belongs to the Argonaute/Piwi family, with versatile regulatory functions such as gene expression, stem cell maintenance, spermatogenesis, etc. Our study reveals novel functions for PIWI proteins in regulating spermatogenesis in *Eriocheir sinensis*. Knocking down *Piwis* enhanced the proliferation and differentiation of spermatogonia, potentially via regulation of the Wnt-signaling pathway. Based on these findings, we propose a model wherein PIWIs maintain the normal spermatogenic cycle by suppressing excessive spermatogonia proliferation and differentiation. These results provide a foundation for exploring PIWI functions in *E. sinensis* and other crustaceans. In a parallel context, our findings in *E. sinensis* offer new insights into the mechanism of *Piwis* in regulating spermatogenesis.

**Abstract:**

The roles of PIWI in mammalian spermatogenesis have been well-studied but are largely unknown in invertebrates such as the Chinese mitten crab (*Eriocheir sinensis*), which produces non-flagellar sperm. Here, we demonstrate that knockdown of PIWIs significantly promotes the proliferation of spermatogonia and the transformation into spermatocytes. Expression of PIWIs in HEK 293T significantly inhibits cell proliferation through the Wnt-signaling pathway. PIWIs suppress transcriptional activity of the Wnt pathway to down-regulate Cyclin D and Cyclin E by inhibiting β-catenin and the phosphorylation of β-catenin at Ser552. The intracellular structure of the adherens junction is destroyed by PIWIs due to downregulated α-catenin, β-catenin, and ZO1. Overall, our results suggest that PIWIs regulate spermatogonia self-renewal and differentiation through inhibiting the Wnt-signaling pathway and stabilize the structure of the adherens junction by regulating the expression and location of α-catenin, β-catenin, and ZO1 in *E. sinensis*, which are different from the functions in mammals. Our findings revealed novel functions and molecular mechanisms of PIWIs in regulating spermatogonia self-renewal and differentiation during the Crustacea spermatogenesis.

## 1. Introduction

Germ cells play a crucial role in the continuation of species, as they are responsible for transmitting genetic information to offspring. For males, the formation of mature germ cells is composed of several processes and can be divided into the following three main steps: mitotic, meiotic, and spermiogenesis phases [1,2]. Mitotic proliferation of spermatogonia provides a stem cell pool for spermatogenesis. Meiotic proliferation produces haploid spermatids. And spermiogenesis is the process of maturation of haploid spermatids. These three processes lay the foundation for the production of functional spermatozoa; any mistake in these processes can impair male fertility. As the complex of these processes, many factors are involved in the regulation of these biological processes [3,4]. Among them, PIWI proteins were found to play significant roles in the regulation of germ cell development [5,6].

The *Piwi* (P-element-induced wimpy testis) gene was initially identified in *Drosophila melanogaster*, playing key roles in Germline Stem Cells (GSCs) asymmetric division, and its mutation resulted in the cessation of GSC division [7]. As a member of the Argonaute/Piwi family, PIWI proteins contain two important domains, named the PAZ and PIWI domains, which are highly conserved during evolution [8,9]. In recent years, evidence has accumulated that PIWI has multitudinous roles in regulating the germline cycle [10]. PIWI is required for the formation of primordial germ cells (PGCs) in *Drosophila* [11]. The absence of maternal PIWI significantly reduces the number of PGCs; the number of PGCs was positively associated with the dose of maternal PIWI [11]. In medaka (*Oryzias latipes*), PIWI was also found to regulate PGC migration. Reduced PGCs were observed when *Piwi* was knocked down, and the depletion of *Piwi* significantly changed the distribution of PGCs [12]. The function of PIWI proteins in germline stem cell maintenance is the most clearly illustrated function. Since it was identified in *Drosophila*, PIWI was found to act in the maintenance of germline stem cells in both males and females [8]. In mice, MILI was required for germline stem cell self-renewal. Depletion of MILI resulted in sterility since no post-meiotic germ cells were found in adult *Mili*-null mice; the expression pattern of spermatogenic stage markers indicated that spermatogenesis was arrested at early stages of meiosis [13]. However, further study found that few spermatogonia were developing at this stage, and the meiotic rate was significantly reduced in *Mili*-null mice at 8 days postpartum, which indicated that MILI was required for spermatogonial stem cell division [14]. PIWI proteins also participated in the regulation of spermatogenic meiosis. Similarly to MILI, the mutation of MIWI and MIWI2 in mice abolished male fertility. No elongated spermatid was found in Miwi^null^ mice, and spermatogenesis was arrested at the round spermatid stage [15]. Prior to the round spermatid stage, drastic apoptosis was found in spermatocytes in *Miwi* mutants [15]. Depletion of MIWI2 showed no haploid germ cells; the spermatogenesis in Miwi2-deficient mice was arrested at the prophase of meiosis I, to be more exact, at the leptotene stage, as only a few mutant spermatocytes reached zygotene, and no pachytene spermatocytes were observed [16]. Two PIWI proteins were found in zebrafish: Ziwi and Zili [17,18]. Zili was also found to function during meiosis. The number of germ cells was reduced in *Zili* mutant fish at the age of 6 weeks without the development of differentiated germ cells [18]. The maturation of spermatids undergoes several biological processes, such as nuclear condensation, which are driven by the transition of histone to protamine [19]. Human PIWI (HIWI) participated in the transition regulation of histone to protamine. HIWI elimination during spermiogenesis was shown to be responsible for the ubiquitination of histones [20], and MIWI was also found to regulate spermiogenic mRNAs [21]. On one hand, MIWI was involved in the elimination of massive mRNA during late spermiogenesis [22]. On the other hand, MIWI also stabilized and activated the translation of the mRNA essential for functional sperm formation [23,24]. The above studies indicate the complex and diverse roles of PIWI proteins in regulating spermatogenesis. During the spermatogenesis process of different species, the functions performed by PIWIs also vary.

Similar to mammalian spermatogenesis, the germ cell development of the Chinese mitten crab (*Eriocheir sinensis*) also undergoes mitotic, meiotic, and spermiogenesis phases. However, several specific characteristics during germ cell development are found in this species. Compared to mammalian round seminiferous tubules, the morphology of seminiferous tubules in crabs seems to be irregular. The seminiferous tubules in the crab can be divided into three parts: germinal, transformation, and evacuation zone [25]. The germinal zone represents one side of the seminiferous tubules, the evacuation zone is the lumen of the tubules, and the transformation zone fills the region between the germinal and the evacuation zone [25]. As the development of germ cells progresses, the germ cells will translocate from the germinal to the evacuation zone. During mammalian spermiogenesis, the most remarkable change is the condensed nucleus with histone to protamine exchange, while uncompacted chromatin is found in crustacean sperm [26]. The differences in the reproductive systems and germ cell development between mammals and crustaceans may indicate different roles of PIWI in mammals and crustaceans.

In our previous study, we found that a lack of PIWIs increased the ratio of abnormal mature sperm in the testis and reduced the apoptotic gene transcriptional level in *E. sinensis* [27]. In the present study, we interfered with *Piwis* in the crabs using double-stranded RNA (dsRNA). The knockdown significantly promoted the proliferation of spermatogonia and their development into spermatocytes. The expression of PIWIs from *E. sinensis* significantly suppressed cell proliferation in HEK 293T cells. PIWIs also downregulated the expression of Cyclin D1, Cyclin D2, Cyclin E1, and Cyclin E2, leading to a cell cycle arrest at the G0/G1 phase. In addition, we found that the regulatory effect of PIWIs on cell proliferation was mediated through the β-catenin-dependent Wnt-signaling pathway. Furthermore, PIWIs markedly reduced the expression of cell junction proteins. The findings of the present study substantially expand our understanding of PIWI functions in crustaceans, highlighting distinct differences compared to those observed in mammals.

## 2. Materials and Methods

### 2.1. Experimental Animals

Healthy male crabs (*E. sinensis*) with an average weight of 105 ± 13 g were purchased from Chongming Research Base of Shanghai Ocean University. Then, all crabs with intact appendages and good vitality were randomly divided into two parts: one part for the cycle of the seminiferous epithelium analysis (30 crabs) and the other part for the experiment of RNA interference (60 crabs). In the first part, 30 crabs were randomly divided into six groups with 5 crabs in each group. In the second part, 60 crabs were divided into four groups with 15 crabs in each group. All the crabs in 1 group were cultured in a plastic box with flowing oxygen at 25 °C. They were fed commercial food once per day, and water was changed daily to maintain the conditions of water quality. Before treatment, the crabs were fed in the feeding room for about a week, and we made sure they adapted to the new breeding environment.

### 2.2. RNA Interference

RNA interference was performed using dsRNA. The dsRNA against three *Piwis* was prepared according to previous reports [27]. dsRNA against *Gfp* was used as a negative control. dsRNA against *piwi1*, *piwi2*, and *piwi3* were used as treatment groups. dsRNA for all four groups was injected into crabs through the first walking leg at a ratio of 2 μg/g body weight (200 μg dsRNA in 200 μL PBS for every crab). All dsRNA was injected every third day, reaching a total of 5 injections. Two days after the fifth injection, testes from the crabs were collected for further analysis.

### 2.3. RNA Extraction and Real-Time PCR

After dsRNA injection, the testes of the crabs were collected. Testes were homogenized in RNAiso Plus reagent (Takara, Beijing, China), and total RNA was extracted according to the manufacturer’s protocol. For RNA from cells, the cells were trypsinized with 0.25% trypsin. After centrifugation, the cells were then resuspended in RNAiso Plus and followed by the same steps applied for the testes RNA extraction. The quality and quantity were measured using a Nano-1000 Micro-spectrophotometer (Allsheng, Hangzhou, China); only the RNA with a ratio of A260/A280 around 1.8–2.0 was used for further analysis. First-Strand cDNA was synthesized using PrimeScript™ RT Master Mix (Takara, Beijing, China). For a 10 μL reaction, 500 ng RNA was used. The cDNA was diluted five times before the Real-Time PCR reaction. The Real-Time PCR reaction was performed in a BioRad CFX Connect Real-Time System (BioRad, Berkeley, CA, USA) using TB Green^®^ Premix Ex Taq™ (Takara, Beijing, China). The reaction contained 12.5 μL of TB Green Premix Ex Taq, 0.5 μL of forward and reverse primer, 2 μL of diluted cDNA, and 9.5 μL of sterile water. A melting-curve analysis was added according to the manufacturer’s protocol to check the specificity of the primer. The expression of the genes was normalized to *esβ-actin*. The primers used in this study are shown in Table 1. The quantifications of gene expression were measured using the 2^−ΔΔCt^ method. Semi-quantitative PCR was used to check the expression of Piwis in HEK 293T cells; the expression of the genes was normalized to *hβ-actin*. PCR was conducted using 2xFlash Hot Start MasterMix (Dye) (CWBIO, Guangzhou, China). The PCR reaction was performed as follows: denaturation at 98 °C for 10 s, annealing at 55 °C for 5 s, and elongation at 72 °C for 15 s, for 25 cycles. PCR products were separated on 1% agarose gels and stained using 4S Red Plus Nucleic Acid Stain (BBI, Sherbrooke, QC, Canada). Images were recorded using Tanon 4200 (Tanon, Shanghai, China).

### 2.4. Western Blotting

For protein from crab testis, the testes were lysed using RIPA Lysis Buffer (Strong) (Beyotime, Shanghai, China) supplemented with PMSF. The testes were homogenized in RIPA buffer, and the DNA complex in the homogenized solution was destroyed by ultrasonication. As for protein from cells, the media were removed, and the cells were washed with PBS. Then, the cells were lysed using Cell Lysis Buffer for Western or IP (Beyotime, Shanghai, China) supplemented with PMSF. For the phosphorylation analysis, a phosphatase inhibitor cocktail was also added. The supernatant was obtained by centrifugation at 12,000× *g* for 10 min. The concentrations were measured using a BCA Protein Assay Kit (Beyotime, Shanghai, China). Lysates from the testis or cells were separated on SDS-PAGE gels. Western blots were performed according to the methods described in a previous study [27]. After the transfer, the PVDF membrane was blocked using 5% non-fat milk in PBST (PBS with 0.05% Tween 20, *v*/*v*) for 2–4 h. Then, the membrane was incubated with primary antibodies overnight at 4 °C. The antibodies against PIWIs were prepared by our lab; the detailed information can be found in our previous report [27]. The next day, the membranes were washed with PBST thrice and then incubated with respective HRP-conjugated secondary antibodies for 60 min at room temperature. Image development was performed using a Tanon 5200 chemiluminescent imaging system by applying BeyoECL Moon (Beyotime, Shanghai, China). All the primary antibodies used for the cell samples were brought from the Beyotime Biotechnology, Shanghai, China. The dilution ratio of the primary antibodies was 1:2000. The blot values were measured by ImageJ v1.8.0.

### 2.5. EdU Incorporation Assay

EdU was used both in vivo and in vitro. For crabs, EdU was injected into the crab from the base of the fourth walking leg at a ratio of 20 mg/kg body weight according to the instructions. After injection of EdU for 2 h, 3, 6, 9, 12, and 15 days, the crab testes were collected for the analysis of the crab spermatogenic cycle. For the RNA interference assay, crabs were injected with EdU two hours before their sacrifice. The collected testes were fixed in 4% PFA overnight at 4 °C and were embedded in paraffin and sectioned at 8 μm. The click reaction was used to label the EdU in the tissues according to the protocol of BeyoClick™ EdU Cell Proliferation Kit with Alexa Fluor 555 (Beyotime, China). The section was immersed in xylene to remove the paraffin. Then, the section was incubated with the click reaction solution for 30 min at room temperature. After washing with 3% BSA in PBS thrice, the section was analyzed under a laser scanning confocal microscope FV3000 (Olympus, Tokyo, Japan). The EdU incorporation assay was also used in vitro for cell proliferation analysis. EdU was added to the cell culture medium at a final concentration of 10 μM. After 1.5 h of culture, cells were trypsinized with 0.25% trypsin and fixed in 4% PFA, then permeabilized using 0.3% Triton X-100 in PBS. EdU immunostaining was conducted according to the protocol of the BeyoClick™ EdU Cell Proliferation Kit with Alexa Fluor 488 (Beyotime, China). EdU-positive cells were counted using Cytoflex (Beckman Coulter, Brea, CA, USA).

### 2.6. Cell Culture and Expression of PIWIs

The full length of PIWI1 was cloned using the primer containing BamH I and Xba I. The full lengths of PIWI2 and PIWI3 were synthesized in Generay (Shanghai, China). Then, the expression fragments were constructed into pCMV-N-flag and pEGFP-c1, respectively. The expressed PIWI 1, 2, and 3 were referred to as OE1, OE2, and OE3 in this study, respectively. Two main domains of PIWI were also cloned using the primers in Table 1, and they were constructed into pEGFP-c1 using ClonExpress II One Step Cloning Kit (Vazyme, Nanjing, China), which we marked as 1PAZ, 2PAZ, 3PAZ (for the PAZ domain), 1PI, 2PI, and 3PI (for the PIWI domain). Recombinant pEGFP-c1 plasmids were used for the immunofluorescence analysis, while the other analyses used the recombinant pCMV-N-flag plasmids. Recombinant plasmids were transformed into TOP10 competent cells (Weidi, Shanghai, China). An EndoFree Plasmid Midi Kit (CWBIO, Guangzhou, China) was used to prepare the endotoxin-free plasmid for cell transfection. HEK 293T cells were cultured using the DMEM media supplemented with 10% FBS and antibiotics in the desired plates. After the cells grew to confluency of 70–80%, endo-free recombinant plasmids were transfected into the cells using Lipo8000 (Beyotime, Shanghai, China), according to the manufacturer’s protocol. The cells were collected for further analysis after being transfected with recombinant plasmids for 48 h.

### 2.7. Immunofluorescence

HEK 293T cells were sub-cultured into 24-well plates, which contained a sterile coverslip inside. Cells were transfected with the recombinant pEGFP-c1 plasmids when the confluency reached 50%. After transfection for 48 h, the medium was removed, and the cells were washed with PBS twice. Then, the cells were fixed with 4% PFA for 30 min and permeabilized using 0.3% Triton X-100 in PBS. After washing with PBS thrice, the cells were blocked using 1% BSA in PBS for 60 min at room temperature. Following blocking, the cells were incubated with primary antibodies at 4 °C overnight. The next day, the cells were incubated with the respective secondary antibodies after washing with PBS thrice using Alexa Fluor 555-labeled goat anti-mouse IgG or Alexa Fluor 555-labeled goat anti-rabbit IgG (Beyotime, Shanghai, China) for 60 min at room temperature in the dark. Tubulin was incubated using Tubulin-Tracker Red (Beyotime, Shanghai, China) with a dilution ratio of 1:100. The nucleus was stained with DAPI. Then, coverslips were mounted on glass slides using an Antifade Mounting Medium (Beyotime, Shanghai, China). Images were observed under a laser scanning confocal microscope FV3000 (Olympus, Tokyo, Japan). All the primary antibodies used for the cell samples were bought from Beyotime Biotechnology, China. The dilution ratio of the primary antibodies was 1:50.

### 2.8. Cell Cycle Analysis

The cells were grown in 12-well plates and transfected with the recombinant pCMV-N-flag plasmids when the confluency reached 70%. After 48 h, the cells were trypsinized with 0.25% trypsin and collected by centrifuging at 1000 rpm for 5 min. Then, the cells were washed with cold PBS, fixed with 70% cold ethanol overnight after another round of centrifugation. The next day, ethanol was removed after centrifugation. The ethanol residue was washed with cold PBS. Then, the cells were treated with RNase and stained with Propidium at 37 °C for 30 min using the Cell Cycle and Apoptosis Analysis Kit (Beyotime, Shanghai, China). Cytoflex (Beckman Coulter, California, USA) was used to detect any fluorescence. Ratios of cells in different cell cycle phases were analyzed using FlowJo V10.

### 2.9. TCF Reporter Assay

Transcriptional factor TCF is the downstream factor of the canonical Wnt-signaling pathway. The transcriptional activity of TCF represents the activated level of the canonical Wnt-signaling pathway. We used the TOP Flash plasmid (Millipore, Burlington, MA, USA) to analyze the transcriptional activity of TCF, as it contains two sets of three copies of the TCF binding site upstream of the Firefly Luciferase open reading frame. The pRL-SV40 is used as an internal control reporter, which contains a cDNA encoding Renilla luciferase. HEK 293T was seeded in the 12-well plates, and PIWI recombinant plasmids were co-transfected with TOP-flash and pRL-SV40 plasmids after 24 h at a ratio of 3:2:1. The media were changed after 4 h. After 48 h of transfection, the media were removed, and the cells were washed with PBS. Then, 100 μL of the Firefly Luciferase Reporter Gene Assay Cell Lysis Buffer (Beyotime, Shanghai, China) was added to the cells. The cell lysate was centrifuged at 12,000× *g* for 10 min, and the supernatant was collected for further experiments. Luciferase activity was detected by Dual Luciferase Reporter Gene Assay Kit (Beyotime, Shanghai, China). The cell lysate, the substrate luciferin, and coelenterazine were dissolved to room temperature. The cell lysate (20 μL) was added to the black cell cluster plates, and 100 μL of the luciferin buffer was added to the cell lysate and incubated at room temperature for 5 min. The relative light unit was measured with VICTOR Nivo (Perkin Elmer, Shanghai, China). After luciferin, 100 μL of the coelenterazine buffer was added. The relative light unit was measured again. The relative luciferase activity was calculated as Firefly luciferase activity/Renilla luciferase activity.

### 2.10. Statistical Analysis

All data in this study were presented as means ± SD (n = 3). The homogeneity of data variance was examined using Levene’s test. The statistical differences were calculated via an unpaired two-tailed Student’s *t*-test using SPSS Statistics 22 (IBM, New York, NY, USA). A *p*-value < 0.05 was considered statistically significant, and a *p*-value < 0.01 indicated an extremely significant change. Graphs in this study were made using GraphPad Prism 9 (GraphPad, Boston, MA, USA). Adobe Illustrator CC 2018 was used to construct the figures.

## 3. Results

### 3.1. Effects of PIWI Deficiency on Spermatogonia Proliferation and Differentiation in E. sinensis

**Figure 1:**
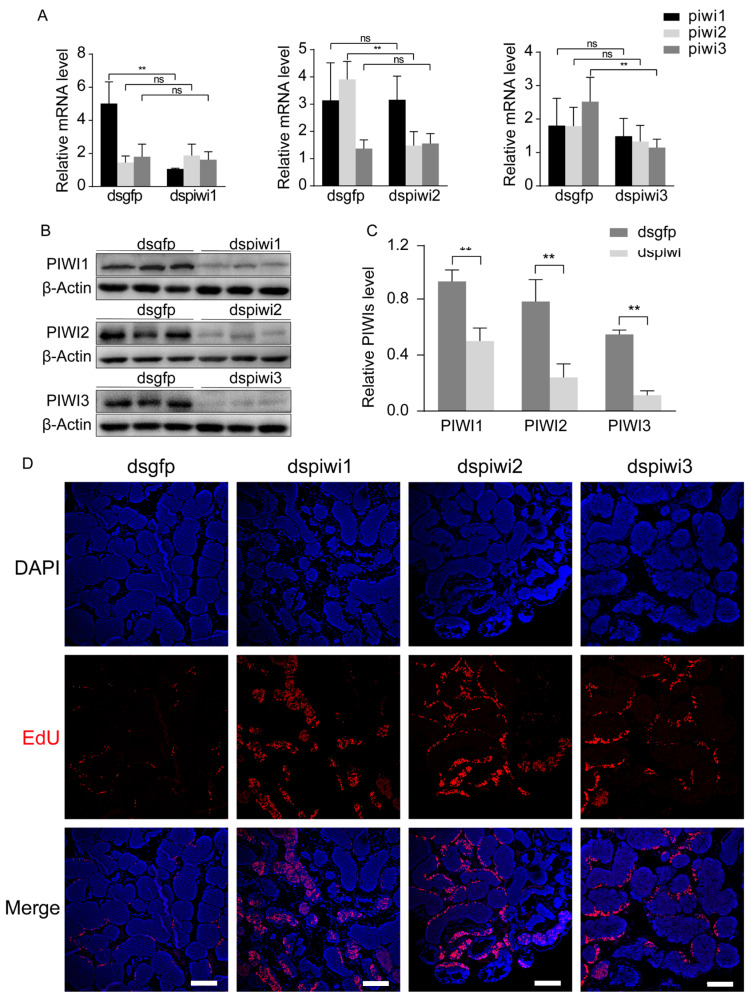
PIWIs regulate the transformation of spermatogonia to spermatocytes in *E. sinensis*. (**A**) Transcriptional level of *Piwis* in the testis of *E*. *sinensis* injected with dsRNA specific to *Piwi1-3*, respectively. (**B**,**C**) Western blot analysis and quantification of PIWIs in *E*. *sinensis* testes injected with dsRNA specific to *Piwi1-3*, respectively. The integrated density was measured using ImageJ. Crabs in the control group were injected with dsRNA against *Gfp*. The actin mRNA and actin protein from crabs were set as an internal control. Three crabs (n = 3) were used in each group. Significant differences were analyzed with an unpaired two-tailed Student’s *t*-test. “**” indicates *p* < 0.01. “ns” indicates no significant change. (**D**) Immunostaining of EdU in the testes of *E. sinensis* injected with dsRNA. Testis samples were collected 2 h after the injection of EdU. Scale bar = 200 μm.

To investigate the functions of PIWIs during spermatogenesis in *E. sinensis*, we constructed specific dsRNA to knock down *Piwis*. As *Piwis* were mainly expressed in the testes, we just checked the expression of *Piwi* genes and PIWI proteins in the testes. After injection of dsRNA, the transcriptional levels of *Piwis* were significantly reduced compared to the control group, where dsRNA specific to *gfp* gene from *Aequorea Victoria* was injected (Figure 1A). The dsRNA against *Piwi1* did not have any effects on *Piwi2* or *Piwi3*. The same results were observed in the knockdown of *Piwi2* and *Piwi3* (Figure 1A), which indicated that specific dsRNA against *Piwi* significantly reduced the transcriptional level of the corresponding *Piwi* without affecting others. As the downregulation of *Piwi* genes occurred, the dsRNA injection also significantly reduced the protein level of PIWIs (Figure 1B,C). Therefore, we successfully inhibited the expression of *Piwis* and PIWIs in *E. sinensis* testes by using dsRNA injection.

To investigate the role of PIWIs during spermatogenesis in *E. sinensis*, we should be familiar with the spermatogenic cycle. So, we first investigated the spermatogenic cycle using an EdU incorporation assay. EdU can be incorporated into germ cells during DNA replication, which happens at the beginning of mitosis and meiosis. The time point at which the EdU signal can be first detected in spermatocytes, spermatids, or spermatozoa represents the duration required for spermatogonia to develop into germ cells at the corresponding stage. We checked the EdU signaling every 3 days after we injected the EdU, as shown in Supplemental Figure S1. The EdU signaling could be detected in the spermatocytes after being injected with EdU for 2 h, 3, 6, 9, 12, and 15 d. We could even detect EdU in spermatocytes 2 h after EdU injection and not find the EdU in spermatozoa even after 15 d (Supplemental Figure S1). On day 12, we observed the existence of EdU in spermatids (Supplemental Figure S1). Therefore, spermatogonia of *E. sinensis* could develop into spermatocytes within 2 h, into spermatids in about 12 d, and into spermatozoa in more than 15 d. In this study, we continuously injected dsRNA for 15 days, which covered the entire period required for spermatogonia to develop into spermatids.

PIWIs have been reported to have roles in germline stem cell maintenance and proliferation [9,28]. We speculated that PIWIs in *E. sinensis* participated in the regulation of spermatogonia proliferation. We used EdU incorporation to investigate the effects of *Piwi* knockdown on spermatogonia proliferation. EdU signaling was detected both in *Piwi*-knockdown testes and the control testes. However, the knockdown groups showed much more EdU signaling in both spermatogonia and spermatocytes compared to the control group (Figure 1D).

### 3.2. Localization of PIWIs in HEK 293T Cells

**Figure 2:**
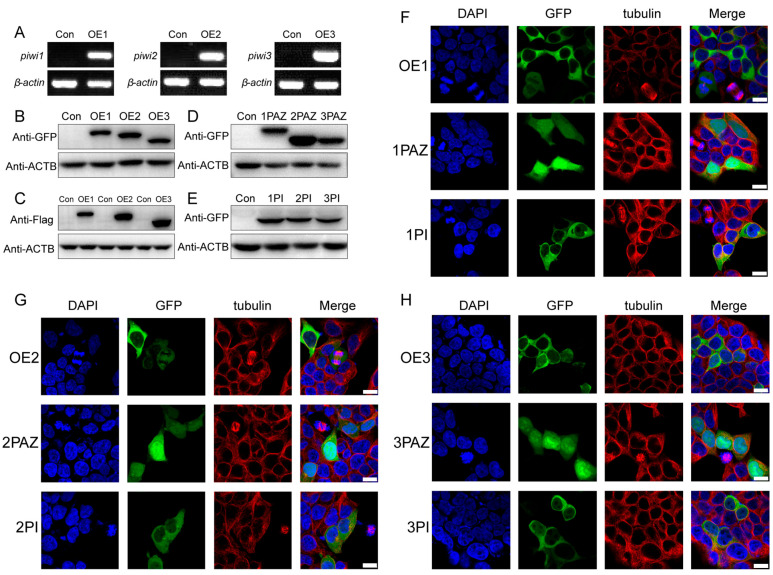
The expression model of PIWIs and PIWI domains from *E. sinensis* in HEK 293T. (**A**) Detection of *Piwis* mRNA in HEK 293T using RT-PCR. β-Actin from humans was set as an internal control. (**B**,**C**) Western blot analysis of PIWIs (constructed into pEGFP-c1 and pCMV-N-flag) from *E. sinensis* in HEK 293T. The actin protein was set as an internal control. (**D**,**E**) Western blot analysis of PIWIs domains (PAZ and PIWI domains constructed into pEGFP-c1) from *E. sinensis* in HEK 293T. The actin protein was set as an internal control. OE1, OE2, and OE3 represented *piwi1*, *piwi2*, and *piwi3*, respectively. (**F**–**H**) Immunostaining analysis on the localization of PIWIs in HEK 293T cells transfected with PIWI expression vectors, which was shown by EGFP (green). The nuclei were stained with DAPI (blue). Tubulin was marked by Tubulin-Tracker Red (red). Scale bar = 15 μm.

As a limitation of cell culture technology in crustaceans, we used HEK 293T cells to investigate the mechanism of the regulation of PIWIs on the proliferation and differentiation of spermatogonia in *E. sinensis*. We constructed PIWI-expressed plasmids, after transfection to HEK 293T cells, and all three *Piwis* could be successfully expressed, with no transcript being found in the control group (Figure 2A). The results of the Western blot showed that the expressed proteins in HEK 293T cells had the same molecular weight as we predicted (Figure 2B,C). We also investigated the potential contribution of two main domains of PIWI, the PAZ and PIWI domains. We expressed these two domains in HEK 293T cells and confirmed their expression via the Western blot (Figure 2D,E).

The localization of PIWIs in *E. sinensis* testes was reported in our previous study, which showed that three PIWIs were expressed in the cytoplasm of spermatogonia, spermatocytes, and spermatids, and PIWI 1 and PIWI 3 were also localized in the cup-like nucleus of spermatozoa [27]. In this paper, we analyzed the localization of PIWIs as well as their PAZ and PIWI domains in HEK 293T cells (Figure 2F–H). Similar to the results in *E. sinensis*, PIWIs were mainly expressed in the cytoplasm, while the PAZ domains were extensively expressed in the cells, including cytoplasm and nuclei, with many more signals being found in nuclei. Compared with the PAZ domain, the PIWI domain was strictly expressed in the cytoplasm. Therefore, PIWIs are mainly expressed in the cytoplasm of HEK 293T cells, and the PIWI domain seemed to contribute more to the localization of PIWIs.

### 3.3. Cell Proliferation Inhibited by Down-Regulating Cyclin After PIWI Expression

**Figure 3:**
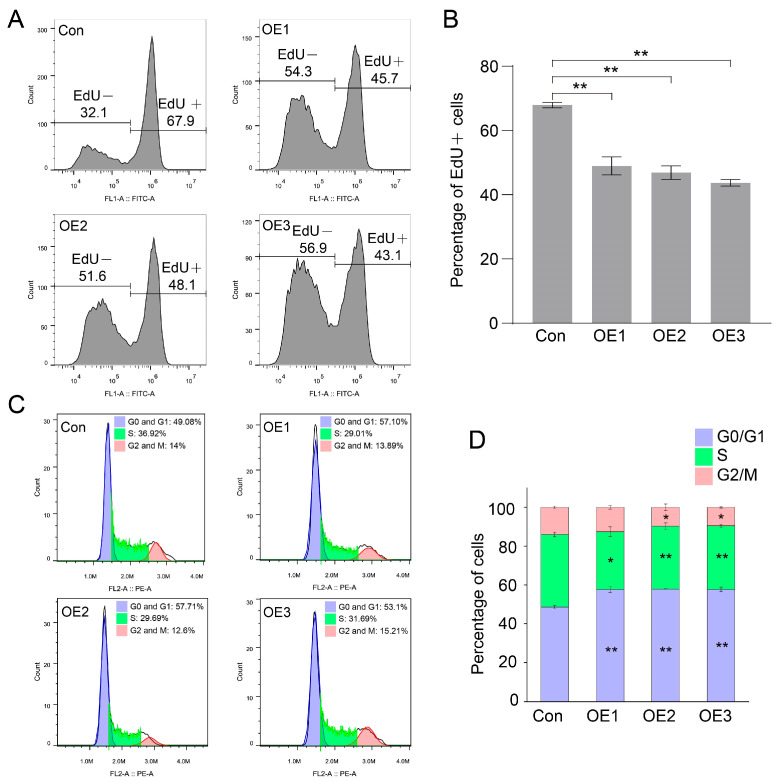
PIWIs from *E. sinensis* inhibited cell proliferation of HEK 293T. (**A**,**B**) EdU incorporation assay and quantification analysis of PIWIs on cell proliferation of HEK 293T by flow cytometry. (**C**,**D**) Cell cycle analysis and quantification analysis in HEK 293T cells transfected with PIWI expression vectors. Triplicate were used in each group (n = 3). Significant differences were analyzed by an unpaired two-tailed Student’s *t*-test. “*” above the columns indicates *p* < 0.05, while “**” indicates *p* < 0.01.

As interference of *Piwis* promoted the proliferation of spermatogonia in *E. sinensis*, we checked the cell proliferation in HEK 293T cells after PIWI expression. The ratio of EdU-positive cells was counted by flow cytometry after EdU addition for 1.5 h. The ratio of EdU-positive cells was 67.9% in the control group (Figure 3A), while it was 45.7%, 48.1%, and 43.1% in the *piwi1-*, *piwi2-*, and *piwi3*-expressed groups, respectively (Figure 3A,B), which indicated that PIWIs significantly decreased the proliferation of HEK 293T cells.

PIWIs regulated cell proliferation by reducing DNA replication. We speculated that the cell cycle should also be changed by PIWIs. Fluorescence-activated cell sorting (FACS) was used to analyze the cell cycle in cells transfected with PIWIs. As we speculated, the cell cycle was significantly altered by PIWI expression (Figure 3C,D). All three PIWIs significantly increased the ratio of cells in the G1 phase and decreased the cells in the S phase. The cell cycle arrest at the G1 phase suggested that fewer cells went through the DNA replication process, and PIWIs reduced the cell proliferation.

**Figure 4:**
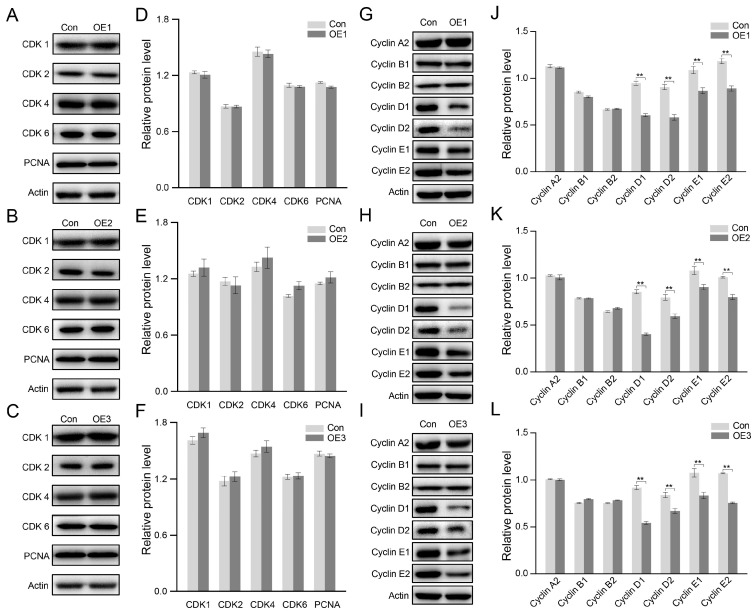
PIWIs from *E. sinensis* inhibit the expression of Cyclin in HEK 293T. (**A**–**F**) Western blot analysis and quantification of cyclin-dependent kinase in HEK 293T cells transfected with PIWI expression vectors. (**G**–**L**) Western blot analysis and quantification of Cyclin in HEK 293T cells transfected with PIWI expression vectors. The actin protein was set as an internal control. The integrated density was measured using ImageJ. Triplicates were used in each group (n = 3). Significant differences were analyzed with an unpaired two-tailed Student’s *t*-test. “**” above the columns indicates *p* <0.01.

Then, the cell cycle-related proteins were checked using the Western blot. PIWI expression did not change the expression of any cyclin-dependent kinase (CDK), including CDK1, CDK2, CDK4, and CDK6 (Figure 4A–F). Same with CDKs, no charge was detected in the expression of the DNA replication marker PCNA. However, the expression of cyclins was significantly changed as the expression of PIWIs (Figure 4G–L). Three PIWIs significantly decreased Cyclin D1, D2, E1, and E2, without altering Cyclin A2, B1, and B2, suggesting that PIWIs inhibited cell proliferation at the G1 phase by down-regulating Cyclin D and Cyclin E.

### 3.4. Canonical Wnt-Signaling Pathway Inhibited After PIWI Expression

**Figure 5:**
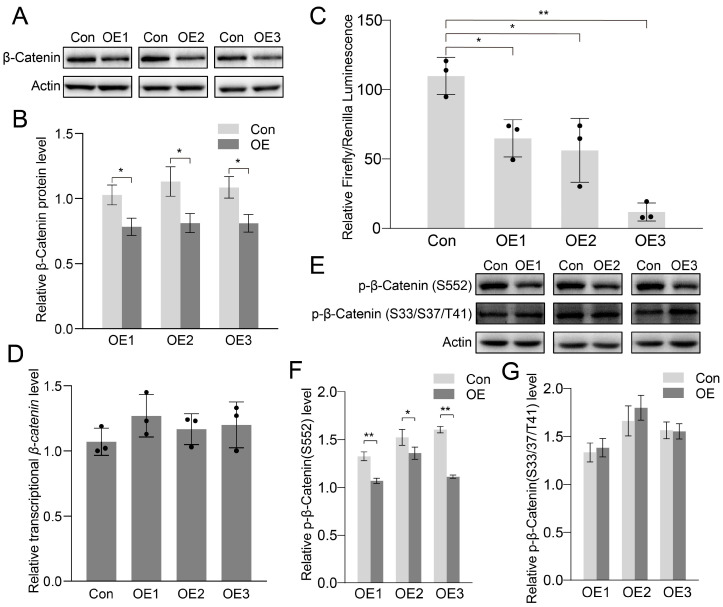
PIWIs from *E. sinensis* inhibit the Wnt-signaling pathway. (**A**,**B**) Western blot analysis and quantification of β-catenin in HEK 293T cells transfected with PIWI expression vectors. (**C**) TCF reporter gene assay in HEK 293T cells transfected with PIWI expression vectors. (**D**) Transcriptional level of β-catenin in HEK 293T cells transfected with PIWI expression vectors. (**E**–**G**) Western blot analysis and quantification of phosphorylation of β-catenin in HEK 293T cells transfected with PIWI expression vectors. The actin mRNA and actin protein were set as an internal control. The integrated density was measured by ImageJ. Triplicate were used in each group (n = 3). Significant differences were analyzed using an unpaired two-tailed Student’s *t*-test. “*” above the columns indicates *p* < 0.05, while “**” indicates *p* < 0.01.

It is reported that Cyclin D is a target gene of the Wnt-signaling pathway [29,30]. We then checked the effects of PIWIs on the Wnt-signaling pathway. The Wnt-signaling pathway is divided into two different types: the canonical pathway (or β-catenin pathway) and the noncanonical pathway [31,32,33]. The most important protein in the canonical pathway is β-catenin. We found that PIWI expression significantly reduced β-catenin (Figure 5A,B). The downregulated β-catenin might have significant effects on the activation of the canonical Wnt pathway. The TOP flash plasmid, which contains the TCF binding site upstream of the Firefly luciferase open reading frame, was used to confirm the activation of the canonical Wnt pathway. As shown in Figure 5C, the luciferase activity in PIWIs was significantly lower than in the control group, suggesting that the canonical Wnt pathway transcriptional activity was inhibited after PIWI expression. Taken together, PIWIs inhibited the canonical Wnt pathway by downregulating β-catenin.

PIWI interacts with piRNA to regulate the expression of genes and transposons at both transcriptional and post-transcriptional levels [34]. We hypothesized that PIWIs regulate β-catenin at the transcriptional level. However, it turned out that the transcriptional level of *β-catenin* was not changed by PIWI expression (Figure 5D). Thus, the downregulated β-catenin might be due to the post-translational modification. As the most important phosphorylation sites, Ser33, Ser37, and Thr41 were responsible for the stability of β-catenin in cells. The phosphorylation of these sites destabilized β-catenin and increased the ubiquitination and degradation of β-catenin [35]. The mutation of these sites increased the stability of β-catenin and resulted in the accumulation in the cytoplasm, which would find entry into nuclei and further activate Wnt signaling [36]. In our study, downregulated β-catenin and Wnt-signaling activation were found in PIWI-expressing cells. This suggests the possibility of higher phosphorylation at Ser33, Ser37, and Thr41. However, Western blotting showed that the phosphorylation at Ser33, Ser37, and Thr41 was similar between control and the PIWI expression groups (Figure 5E,G). However, the phosphorylated β-catenin at Ser552, which could promote Wnt signaling transcriptional activity [37,38], was decreased in the PIWI expression groups than in the control group (Figure 5E,F).

### 3.5. Effects on Regulation of Cell Junction Proteins After PIWI Expression

**Figure 6:**
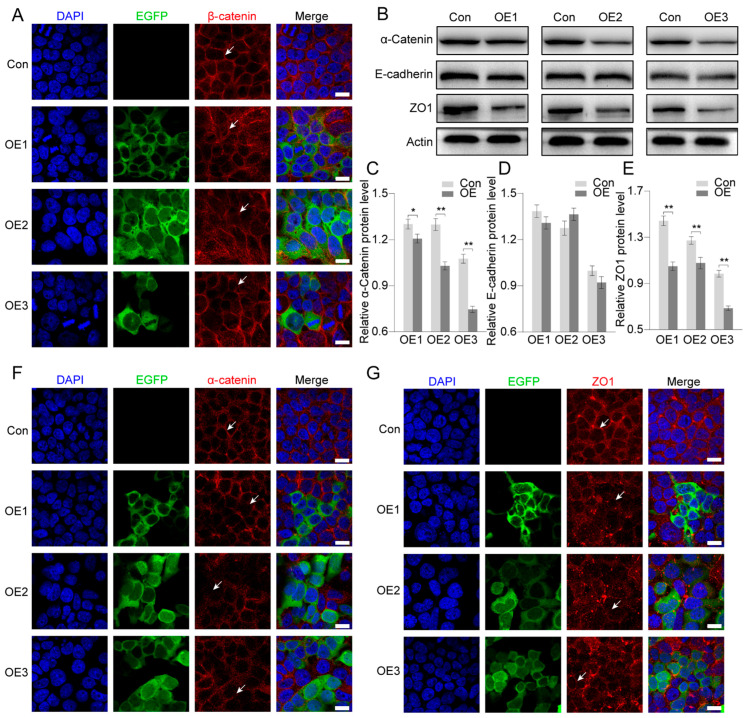
PIWIs regulate adherens junctions in HEK 293T. (**A**) Immunostaining analysis of β-catenin (red) in HEK 293T cells transfected with PIWI expression vectors, which was shown by EGFP (green). The nuclei were stained with DAPI (blue). Scale bar = 15 μm. (**B**–**E**) Western blot analysis and quantification of proteins relative to adherens junctions in HEK 293T cells transfected with PIWI expression vectors. The actin protein was set as an internal control. The integrated density was measured by Image J v1.8.0. Triplicates were used in each group (n = 3). Significant differences were analyzed by an unpaired two-tailed Student’s *t*-test. “*” above the columns indicates *p* < 0.05, while “**” indicates *p* < 0.01. (**F**,**G**) Immunostaining analysis of α-catenin and ZO1 (red) in HEK 293T cells transfected with PIWI expression vectors, which was shown by EGFP (green). The distribution of β-catenin, α-catenin and ZO1 is denoted by white arrows. The nuclei were stained with DAPI (blue). Scale bar = 15 μm.

Except for the important factors in the Wnt-signaling pathway, β-catenin also functions in cell–cell interactions, as it links cadherin to the cytoskeleton [39]. In the present study, β-catenin was significantly inhibited by PIWIs. We speculated that the adherens junction might also be affected by PIWIs. The localization of cell junction proteins indicated significant damage in cell–cell interactions. In the control group, β-catenin was strictly and compactly distributed on the cell membrane, while in PIWI expression groups, it seemed that they were translocated from the membrane to the cytoplasm, which would decrease cell–cell interactions (Figure 6A).

The results of Western blots indicated that the adherens junction protein α-catenin was also significantly changed by PIWI expression (Figure 6B,C). However, E-cadherin was unchanged (Figure 6B,D). As β-catenin and α-catenin were significantly reduced, ZO-1 (zonula occludens-1), a tight junction protein, was also significantly reduced by PIWIs (Figure 6B,E). Immunofluorescence indicated that cells after PIWI expression showed less signaling of α-catenin in the cell membrane compared to control cells (Figure 5F). In the control group, cells had an intact ZO1 signal, but in PIWI-expressed groups, the structure of ZO1 in the membrane was destroyed completely (Figure 6G). These results indicated that PIWIs inhibited cell proliferation and destroyed the cell–cell interaction.

## 4. Discussion and Perspectives

### 4.1. Novel Functions of PIWIs in Regulating Spermatogonia Self-Renewal and Differentiation During E. sinensis Spermatogenesis

Spermatogonia are a type of Germline Stem Cells (GSCs) and are characterized by their capacity for self-renewal and differentiation into spermatocytes. They play a fundamental role in spermatogenic cell development and maintenance throughout life. The *Piwi* genes were initially identified as key regulators of GSC asymmetric division in *D. melanogaster* [7]. PIWI proteins, which exhibit RNA endonucleolytic activity guided by piRNAs, are highly expressed in animal gonads [40]. Early studies using *Piwi* mutants demonstrated that *Piwi* regulates GSC self-renewal and differentiation in different species. Decreased *Piwi* expression in *Caenorhabditis elegans* reduces the proliferation of GSC-equivalent cells [9]. In the *Drosophila* ovary, *Piwi* is required in somatic signaling cells to maintain germline stem cells, such as self-renewal and differentiation [28,41]. However, *Piwi* abnormalities in mammals result in different outcomes compared with those of invertebrates, and their loss leads to male sterility [42,43]. A recent study showed that a deficiency of *Piwi* resulted in severe developmental disorders in spermatogenesis of golden hamsters, such as developmental arrest or cell death beginning after meiosis [44]. Therefore, all these studies have revealed the diverse functions of PIWIs in various biological and developmental processes.

In the present study, much more EdU signaling was detected in both spermatogonia and spermatocytes in each *piwi* knockdown group. That is, more spermatogonia finished the proliferation and differentiation into spermatocytes in the *Piwi* knockdown groups. Therefore, PIWIs suppressed the proliferation of spermatogonia and the transformation of spermatogonia into spermatocytes to maintain the dynamic balance between spermatogonia and spermatocytes during spermatogenesis in *E. sinensis*. Similar studies were conducted in *Penaeus monodon* and *Scylla paramamosain* [45,46]. Both studies found that *piwi2* knockdown led to a significant decrease in the number of sperm cells. Furthermore, the authors held that *Sp-Piwi2* may play an important role in spermatogonia proliferation and differentiation during spermatogenesis in *S. paramamosain.* However, they predicted that *Sp-Piwi2* silencing presumably inhibited spermatogonia self-replication and the transition from spermatogonia to spermatocytes through transcriptome sequencing and differential gene analysis (DEGs), which is exactly in contrast with our results [46]. Therefore, inhibiting spermatogonia self-replication and the transition from spermatogonia to spermatocytes in *E. sinensis* may be *Piwi*’s new function, and further studies are required on the roles of PIWIs in crustacean spermatogenesis.

### 4.2. PIWIs Suppressed Spermatogonia Proliferation and Differentiation Through Wnt Signaling Pathway

PIWI was initially identified in the reproductive system and is predominantly expressed in the gonads of *Drosophila*. Furthermore, it has also been detected in tumor tissues of mammals [47]. In tumor tissues, PIWI interacts with piRNA to regulate cell proliferation, differentiation, and survival [48]. PIWIL2 bound with piRNA-54265 to activate the STAT3 pathway and then promoted cell proliferation in colorectal adenocarcinoma [49]. In non-small cell lung carcinoma, piR-651 increased the expression of Cyclin D1 and CDK4 to promote tumor progression [50], while piR-55490 inhibited cell proliferation in lung cancer cells by targeting the 3′ UTR (untranslated region) of mTOR mRNA [51]. Similarly, piR-39980 targeted RRM2 (ribonucleoside–diphosphate reductase subunit M2) mRNA 3′ UTR to inhibit cell proliferation of HT1080 fibrosarcoma cells [52]. These results suggest that the effects of PIWIs on cell proliferation vary between different piRNAs and different cell types, which raises our concern about the role of PIWI on germ cell proliferation in *E. sinensis*.

In the present study, we found that knockdown of *Piwis* promoted spermatogonia proliferation. To investigate the potential mechanism of the roles of PIWIs in this process, we expressed PIWIs of *E. sinensis* in HEK 293T. Consistent with our conjecture, PIWIs significantly inhibited the cell proliferation of HEK 293T. The Wnt-signaling pathway is a well-known pathway in the regulation of cell proliferation [53]. During spermatogenesis, Wnt5a activates JNK to promote spermatogonial stem cell self-renewal [54]. Wnt3a also positively regulated spermatogonial stem cell proliferation [55]. Wnt/β-catenin signaling is induced by Wnt6 to promote the proliferation of undifferentiated spermatogonia [56]. We checked the effects of PIWIs on Wnt signaling. The analysis of the TCF Reporter Gene showed that the luciferase activity in PIWIs was significantly lower than in the control group, suggesting that the canonical Wnt pathway transcriptional activity was inhibited after PIWI expression. In addition, *Cyclin D* and *Cyclin E* are target genes of the Wnt-signaling pathway, so their expression levels are regulated by the Wnt-signaling pathway. It exactly matches our result that Cyclin D and Cyclin E decreased significantly after the expression of PIWIs from *E. sinensis* in HEK 293T cells.

In this study, we found that PIWIs significantly inhibited the Wnt-signaling pathway by regulating β-catenin, which is consistent with that of HILI in HeLa and HepG2 cells. However, knockdown of HILI in HeLa and HepG2 significantly inhibited β-catenin by regulating the phosphorylation and ubiquitylation of β-catenin, while the overexpression of HILI activated the Wnt-signaling pathway due to the accumulation of β-catenin in the nucleus [57]. In *Drosophila*, PIWI was reduced in the aged niche of germline stem cells. The reduced PIWI derepressed transposons/transposable elements and resulted in the activation of Toll-GSK3 signaling. The activated Toll-GSK3 signaling would further induce degradation of β-catenin [58].

**Figure 7:**
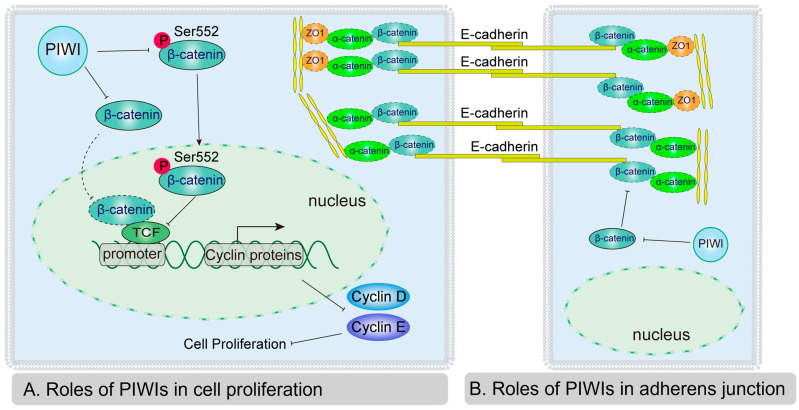
Schematic diagram illustrates the roles of PIWIs in spermatogenesis. Roles of PIWIs in cell proliferation (**A**) and adherens junctions (**B**). PIWIs down-regulate both β-catenin and the phosphorylation of β-catenin at Ser552 to inhibit the transcriptional activity of the Wnt-signaling pathway. Repressed Wnt signaling reduces the expression of Cyclin D and Cyclin E to inhibit cell proliferation, which may inhibit spermatogonia proliferation and transformation of spermatogonia to spermatocytes in *E. sinensis*. Down-regulated β-catenin due to PIWIs also significantly destroys adherens junctions by inhibiting and impairing the localization of α-catenin and ZO1.

Previous studies found that PIWI regulated β-catenin by changing the phosphorylation and ubiquitylation of β-catenin. We investigated the potential mechanism of PIWIs from *E. sinensis* in β-catenin regulation. A well-known function of PIWI is transposons or transposable element repression, as well as mRNA degradation at the guide of piRNA [59,60]. We analyzed the effects of PIWIs on β-catenin mRNA. However, the mRNA level of β-catenin was not affected by the expression of PIWIs from *E. sinensis*, which showed similar results obtained from HeLa and Hep2G [57]. In the canonical Wnt-signaling pathway, the phosphorylation of β-catenin induced by GSK3β and CK1 contributes to the degradation of β-catenin [61]. The level of phosphorylated β-catenin at Ser33/Ser37/Thr41, measured by Western blotting, was unchanged. This indicates that downregulated β-catenin due to the expression of PIWIs does not rely on GSK3. PIWI also had roles in the regulation of translation processes. PIWI interacts with eIF3a, eIF4E, and eIF4G to regulate protein expression by translational activation and repression [16,62,63]. Thus, we speculate that PIWIs from *E. sinensis* may regulate β-catenin at the translational level. Although the phosphorylation of β-catenin at Ser33/Ser37/Thr41 was unchanged by PIWIs, the phospho-β-catenin (Ser552) was significantly downregulated. The phosphorylation of β-catenin at Ser552 induced by PKA (protein kinase A) and AKT is responsible for the activation of the Wnt-signaling pathway [37,38]. Collectively, our results indicated that PIWIs inhibited the Wnt-signaling pathway by directly downregulating β-catenin, as well as the phosphorylation of β-catenin at Ser552. In conclusion, a possible mechanism of PIWI-inhibited spermatogonia proliferation and differentiation was proposed in our study: on one hand, PIWIs directly downregulate β-catenin and decrease the accumulation of β-catenin in nuclei to inhibit Wnt signaling; on the other hand, PIWIs inhibit the phosphorylation of β-catenin at Ser552 and further inhibit the expression of Cyclin D and Cyclin E (Figure 7A).

### 4.3. PIWIs Affect Adherens Junctions by Regulating α-Catenin and ZO1

In addition to functioning in the Wnt-signaling pathway, β-catenin also contributes to the formation of adherens junctions [39,64]. The adherens junction contains E-cadherin, α-catenin, β-catenin, and p120-catenin [65]. E-cadherins in different cells bind with each other through an extracellular domain [66]. α-catenin binds with the cytoplasmic domain of E-cadherin through β-catenin [67,68]. α-catenin can directly link the complex to the cytoskeleton or interact with ZO1 [69]. As the downregulated β-catenin was due to the expression of PIWIs, we also checked the adherens junction changes. When PIWIs were expressed, E-cadherin was unchanged, but the protein levels of α-catenin and ZO1 were significantly decreased. At the same time, the subcellular localization of α-catenin and ZO1 was altered, transitioning from a predominantly membrane-associated distribution to a more diffuse cytoplasmic localization. Therefore, we concluded that PIWIs *E. sinensis* affected the adherens junction through regulation of the expression and localization of α-catenin and ZO1 (Figure 7B). Unfortunately, we were unable to obtain more relevant data on *E. sinensis*; there is still a lot of room for research in the future.

## 5. Conclusions

In conclusion, new functions of PIWIs were found in regulating spermatogenesis in *E. sinensis*. The knockdown of *Piwis* may promote the proliferation and differentiation of spermatogonia in *E. sinensis* by regulating the Wnt-signaling pathway. Abnormal expression of PIWIs destroys the adherens junction by changing the localization or expression of α-catenin and ZO1. Furthermore, we proposed a possible mechanism of PIWIs in suppressing the excessive proliferation and differentiation of spermatogonia to maintain the normal spermatogenesis cycle in *E. sinensis*. These results of the present study could provide important information for further exploration of PIWI’s function in *E. sinensis* and other crustacean species.

## Figures and Tables

**Table 1:** The primer sequences used in the present study.

Gene		Primer Sequence (5′–3′)	Purpose
Piwi1	F	ACACCCCAAAATCCGAGACC	qPCR
	R	CTTGTCCATGCTCCCCTTGT	
Piwi2	F	TTGATGCGCCGATGTATGGA	qPCR
	R	GGATGGTGCTGGTGTATCCC	
Piwi3	F	AAGGCATACCAAGTAGCCGTG	qPCR
	R	TAGCAATGACTCCATGGTCGG	
esβ-actin	F	CGAGGCTACACCTTCACGAC	qPCR
	R	ACGCGGCAGTGGTCATTT	
β-catenin	F	CATCTACACAGTTTGATGCTGCT	qPCR
	R	GCAGTTTTGTCAGTTCAGGGA	
hβ-actin	F	CTCCATCCTGGCCTCGCTGT	qPCR
	R	GCTGTCACCTTCACCGTTCC	
1PAZ	F	TCGAGCTCAAGCTTCGAATTCCAGCACTTGCCTCAATGTAATGG	expression
	R	TTATCTAGATCCGGTGGATCCAGTGGGTGTGACACGGG	
1PI	F	TCGAGCTCAAGCTTCGAATTCGTTGGCAGTCATAATCTTTCCAAC	expression
	R	TTATCTAGATCCGGTGGATCCCTTCACATTCTGACCAATGAGATA	
2PAZ	F	TCGAGCTCAAGCTTCGAATTCGGAGACAGCCTACACCGTCG	expression
	R	TTATCTAGATCCGGTGGATCCGTCTGGGGCGGTGCG	
2PI	F	TCGAGCTCAAGCTTCGAATTCGCTGGTGCTGGTGGTGCTC	expression
	R	TTATCTAGATCCGGTGGATCCTGCCTTGTTGACGATCTGGC	
3PAZ	F	TCGAGCTCAAGCTTCGAATTCCGACACTGTGTACAACCTCCTTCG	expression
	R	TTATCTAGATCCGGTGGATCCGTCTGGGCTGAGGCGTGT	
3PI	F	TCGAGCTCAAGCTTCGAATTCCCTGGTGGTGTGCGTCATCCC	expression
	R	TTATCTAGATCCGGTGGATCCATGGATTGCTTGGCCTGTGATGG	

## Data Availability

All data are included in the main text or Supplementary Material. Further inquiries can be directed to the corresponding author.

## Supplementary Materials

The following supporting information can be downloaded at: https://www.mdpi.com/article/10.3390/biology14101440/s1, Supplemental Figure S1. Cycle of seminiferous epithelium analysis in E sinensis by EdU incorporation assay. EdU was injected to crabs, and the signaling of EdU (red) was detected at 2 h, 3, 6, 9, 12 and 15 d using Confocal Laser Scanning Microscope. Scale bar shows 50 μm. DNA replication happens in spermatogonia, which means that EdU can only be incorporated in spermatogonia. The timing when we can detect EdU signaling in spermatocyte, spermatid or spermatozoa can represent the time required in the development of spermatogonia to the specific stage germ cells. The supplementary Figures S2–S6 are the original full Western blot figures in this study.
