# Supplementary material for: PIWIs Regulate Spermatogonia Self-Renewal and Differentiation by Wnt/β-Catenin Signaling Pathway in Eriocheir sinensis
Source: Biology (Basel). 2025 Oct 18;14(10):1440. doi: 10.3390/biology14101440 (PMC12561773; doi:10.3390/biology14101440)
Supplement: Supplementary file 1 [file biology-14-01440-s001.zip › biology-3869808-WB figures-S2-S6.pdf]

Figure S2B

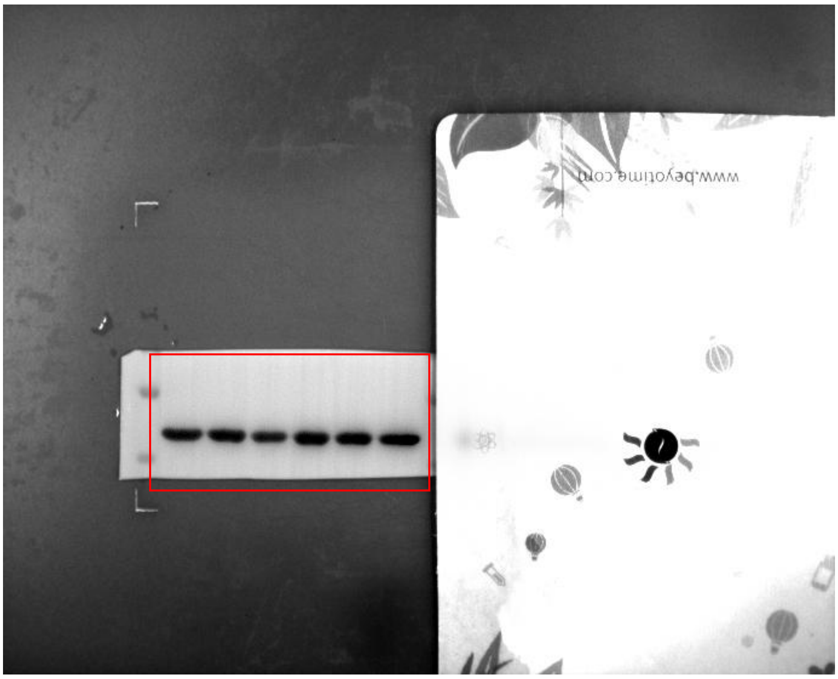

β-Actin (dsgfp 1-3; dspiwi1 4-6)

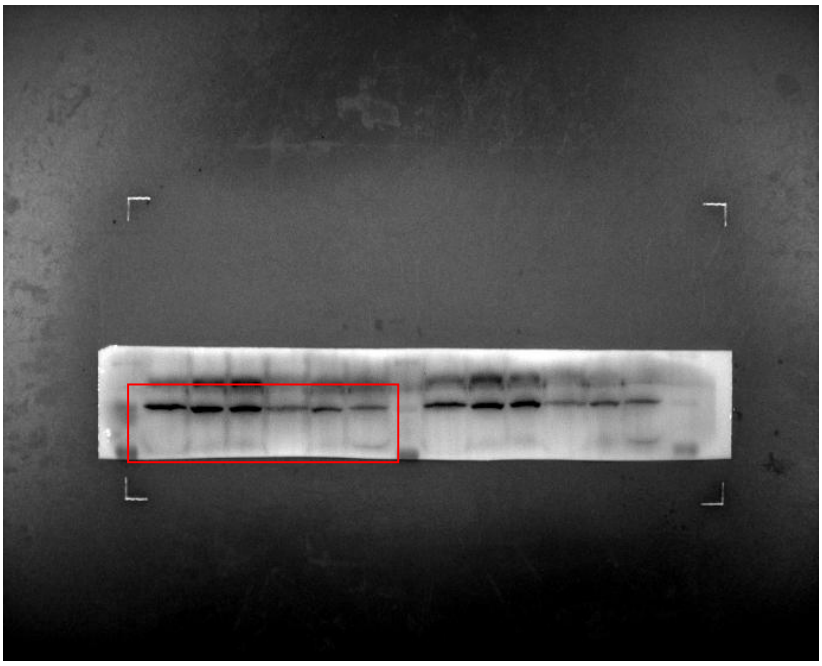

PIWI 1 (dsgfp 1-3; dspiwi1 4-6)

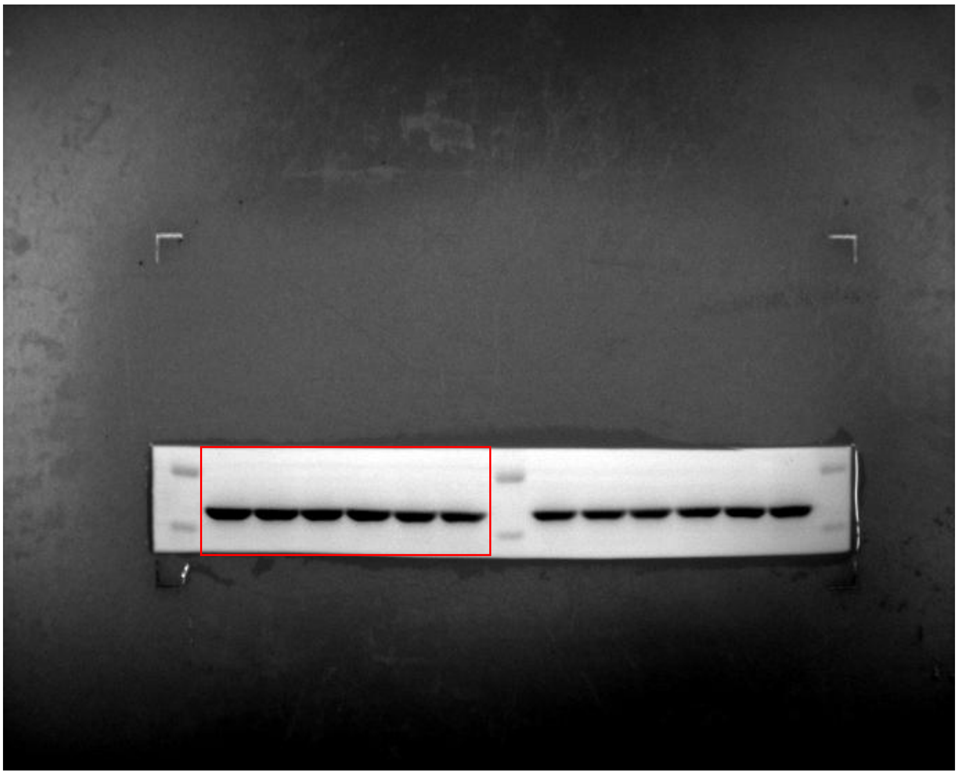

β-Actin (dsgfp 1-3; dspiwi2 4-6)

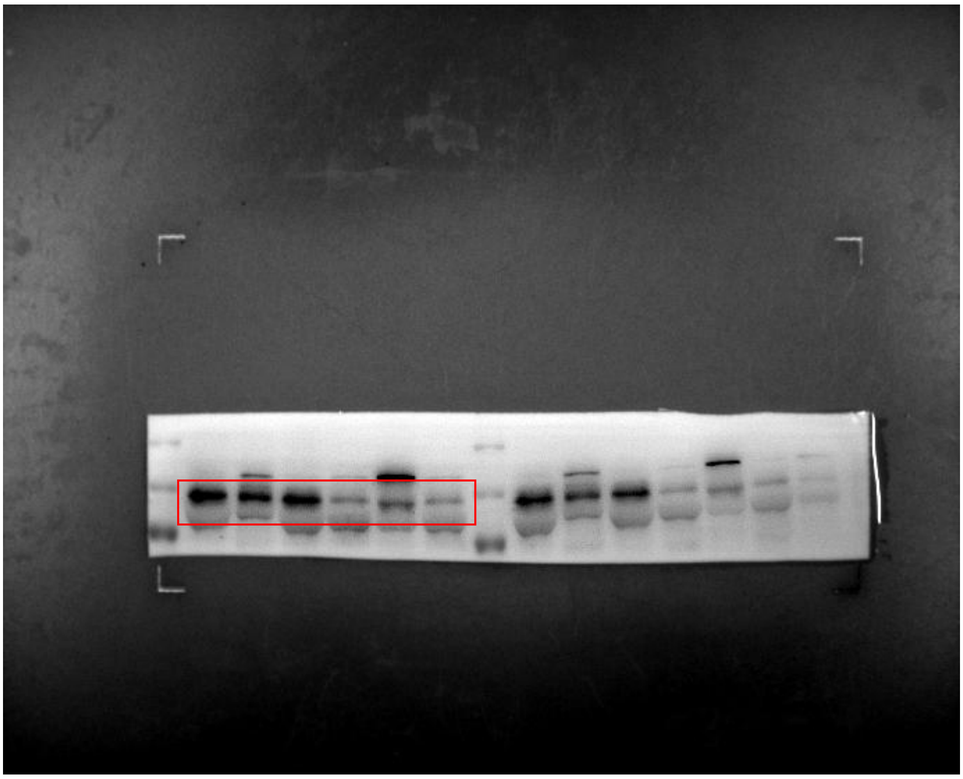

PIWI 2 (dsgfp 1-3; dspiwi2 4-6)

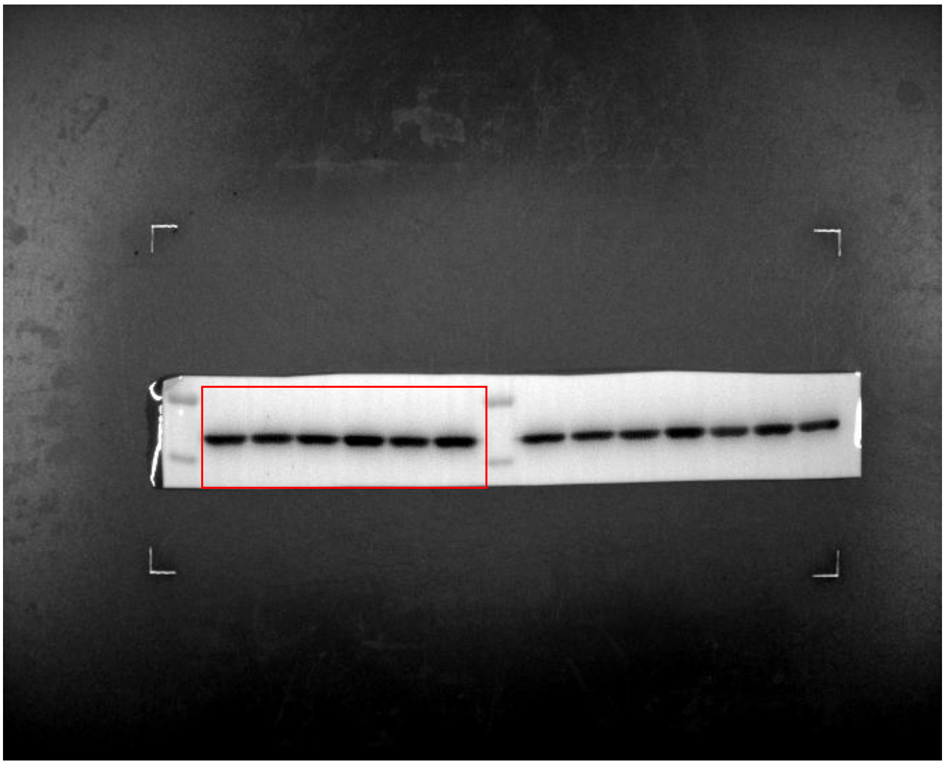

β-Actin (dsgfp 1-3; dspiwi3 4-6)

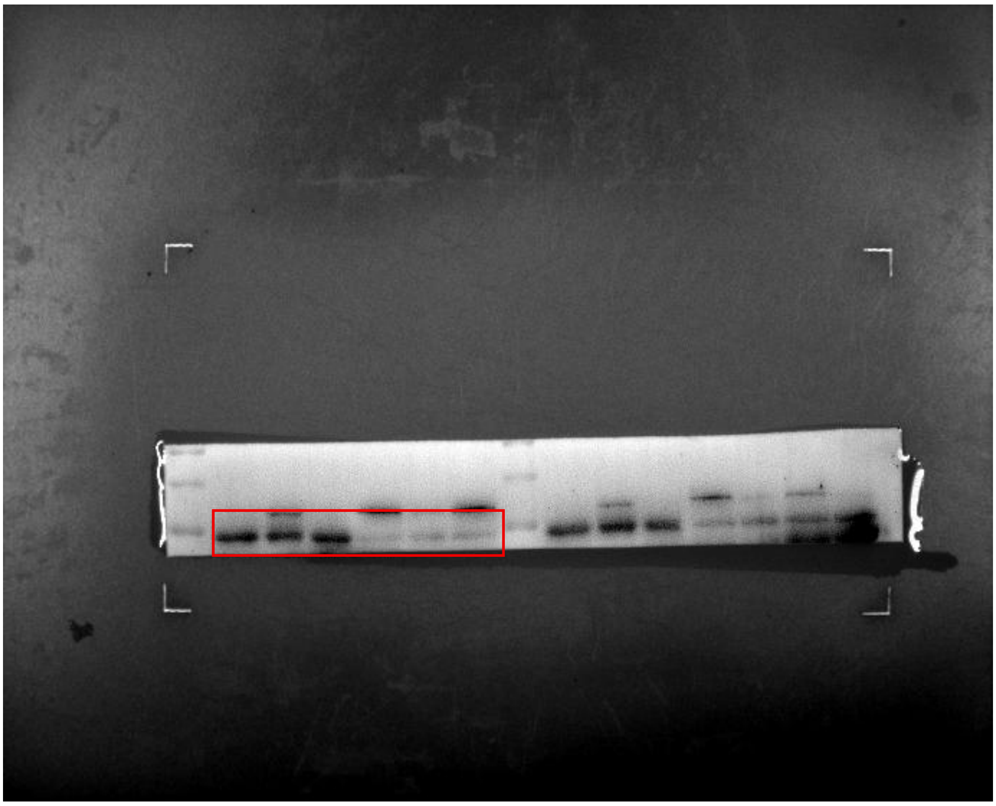

PIWI 3 (dsgfp 1-3; dspiwi3 4-6)

Figure S3

B

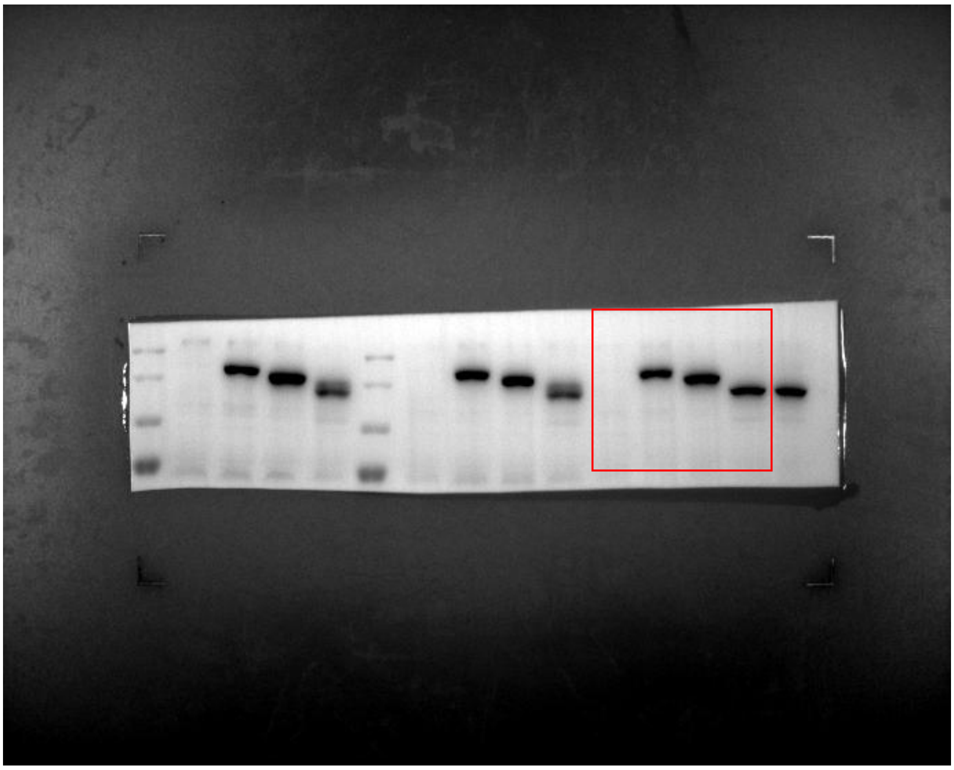

Anti-GFP (Con, OE1, OE2, OE3)

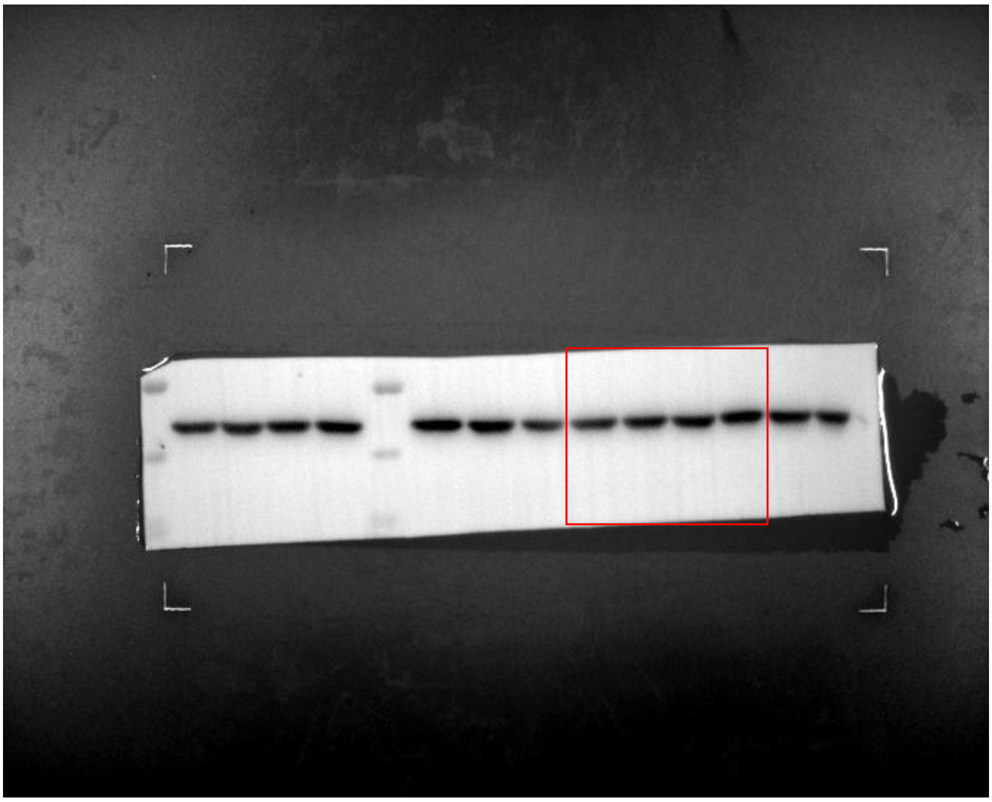

Anti-ACTB (Con, OE1, OE2, OE3)

C

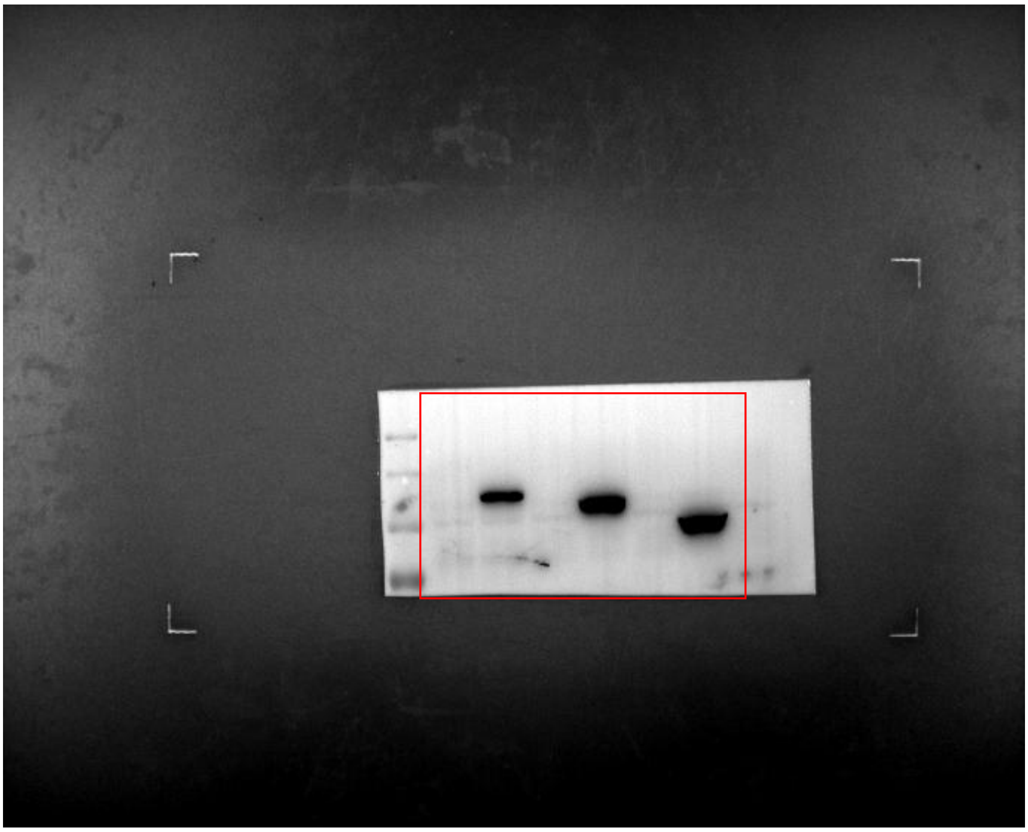

Anti-Flag (Con, OE1； Con, OE2； Con, OE3)

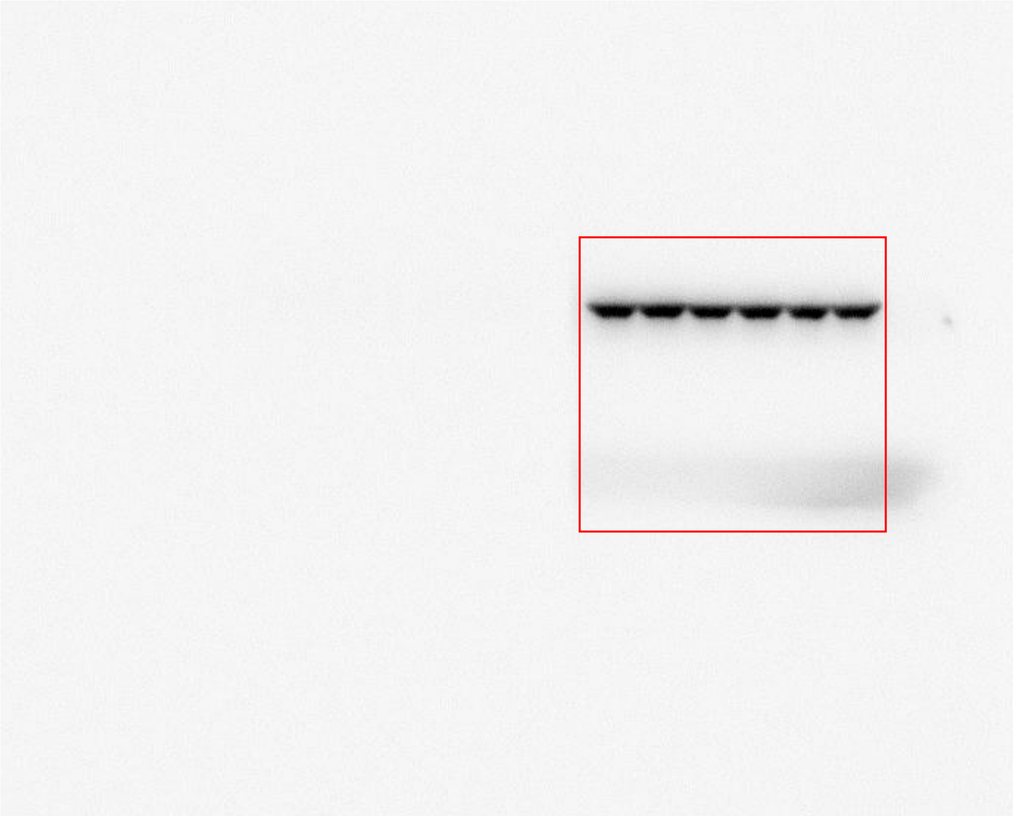

Anti-ACTB (Con, OE1； Con, OE2； Con, OE3)

D

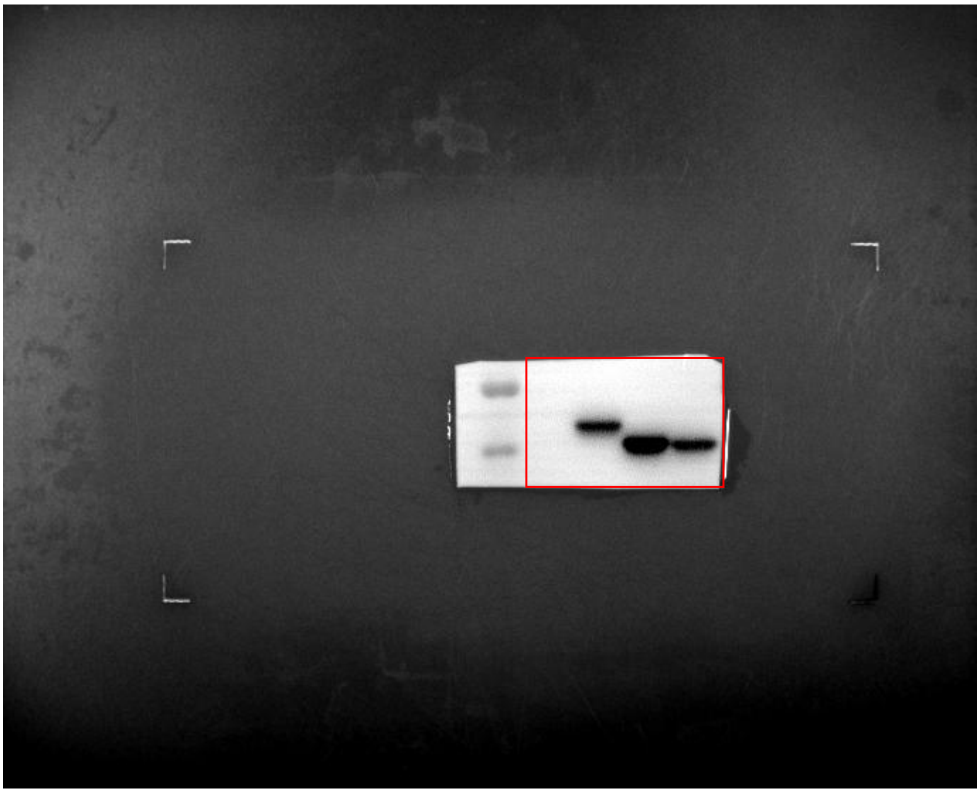

Anti-GFP (Con, OE1； Con, OE2； Con, OE3)

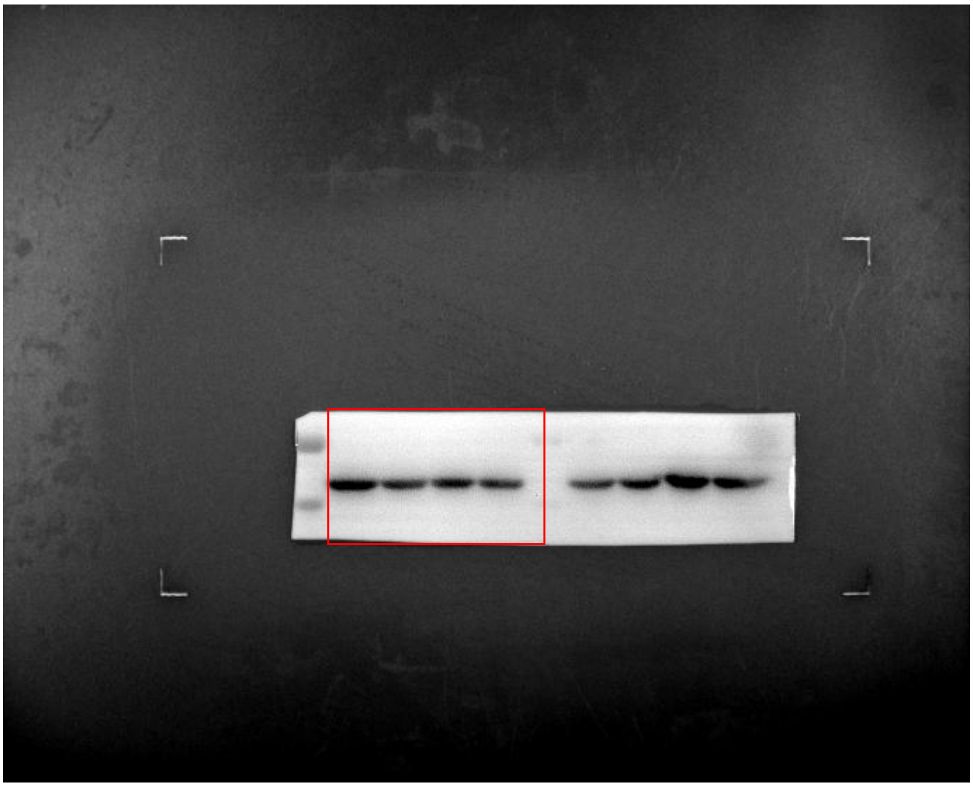

Anti-ACTB (Con, OE1； Con, OE2； Con, OE3)

E

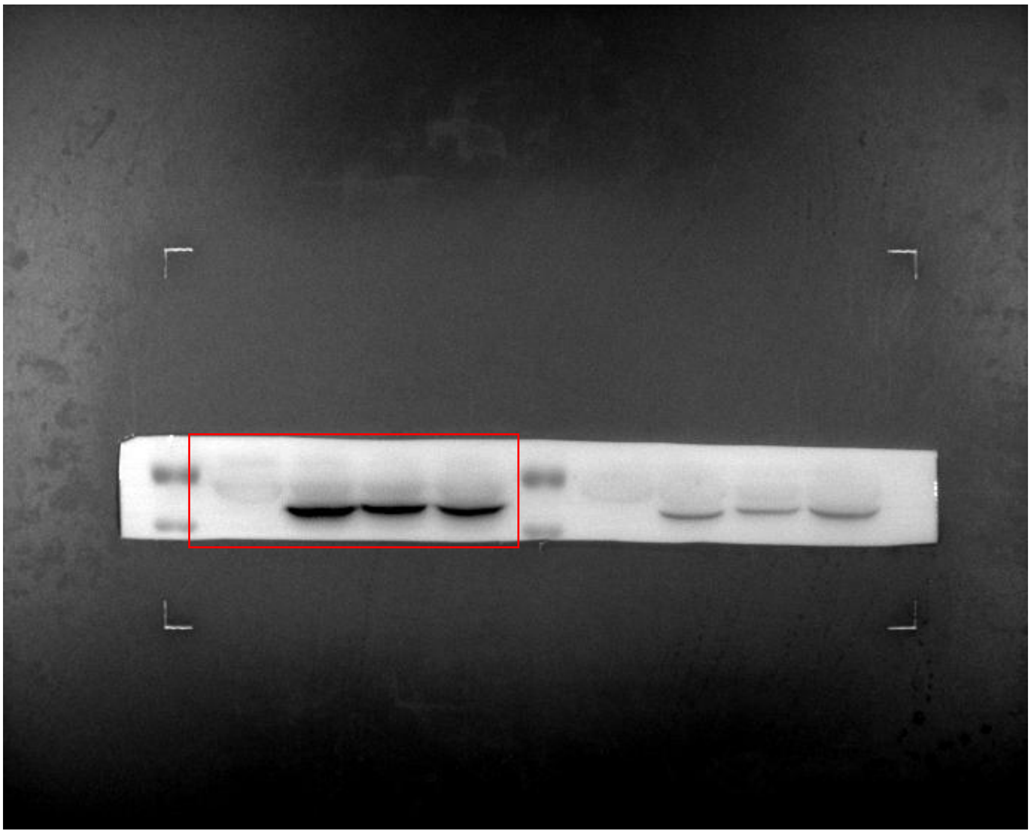

Anti-GFP (Con, OE1； Con, OE2； Con, OE3)

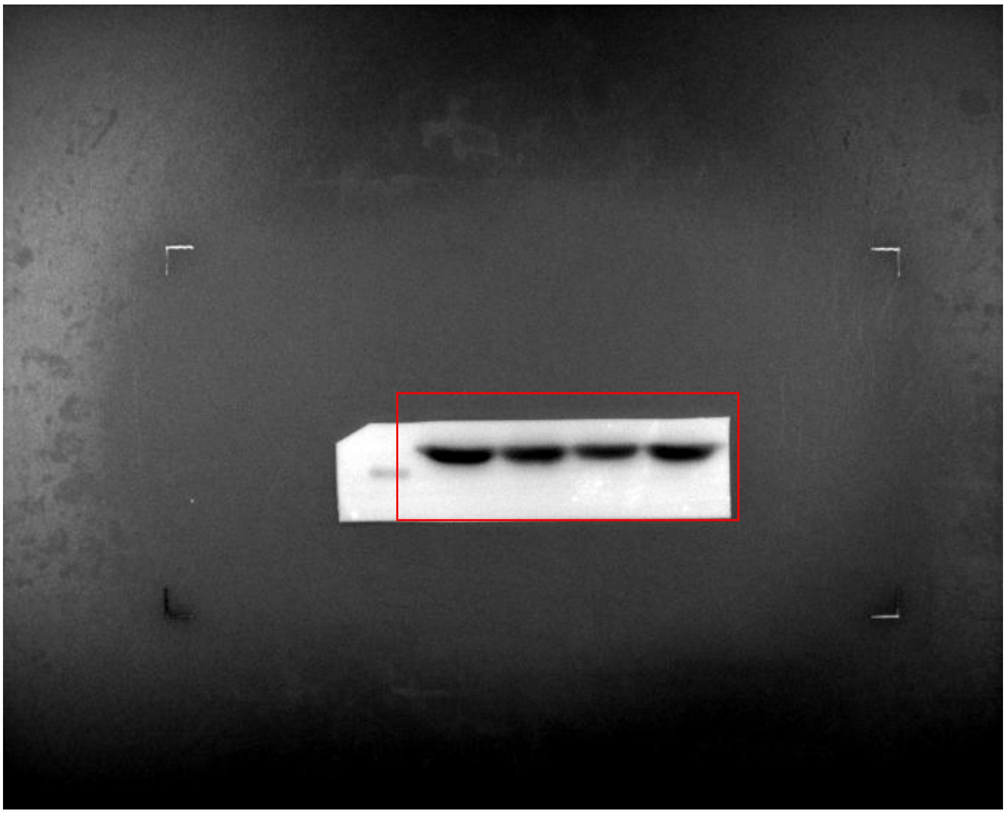

Anti-ACTB (Con, OE1； Con, OE2； Con, OE3)

Figure S4

A-C

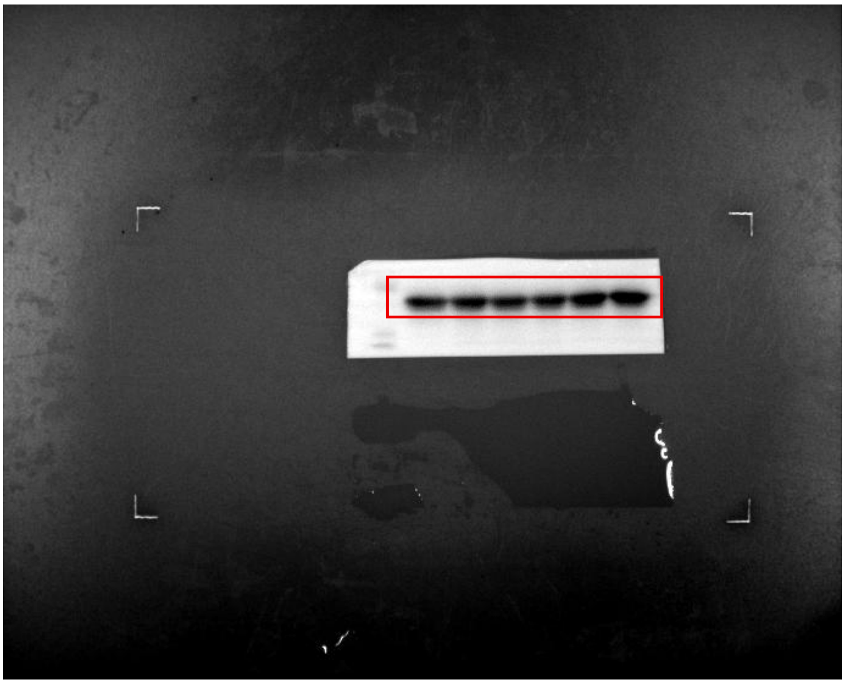

CDK1 (Con, OE)

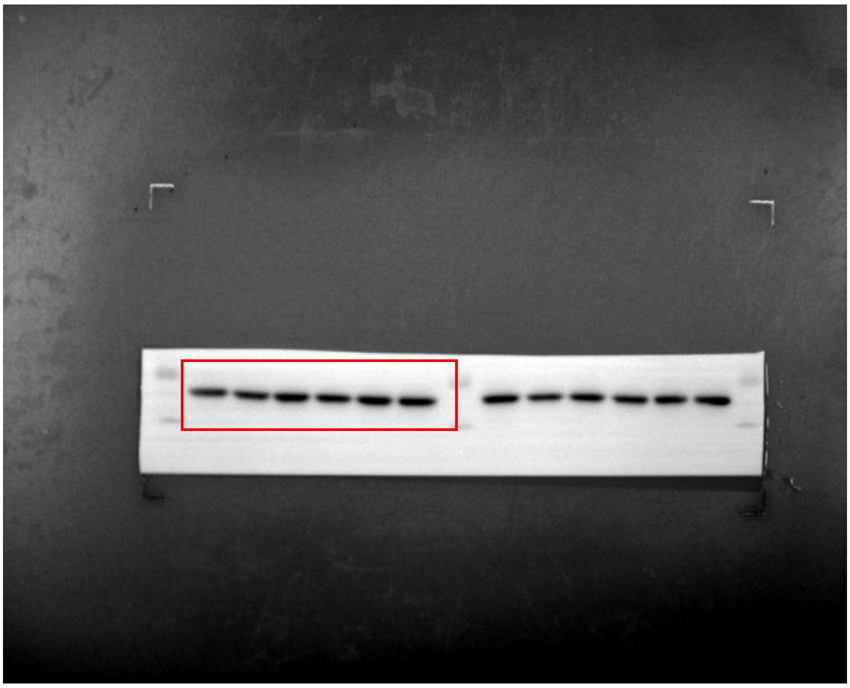

CDK2 (Con, OE)

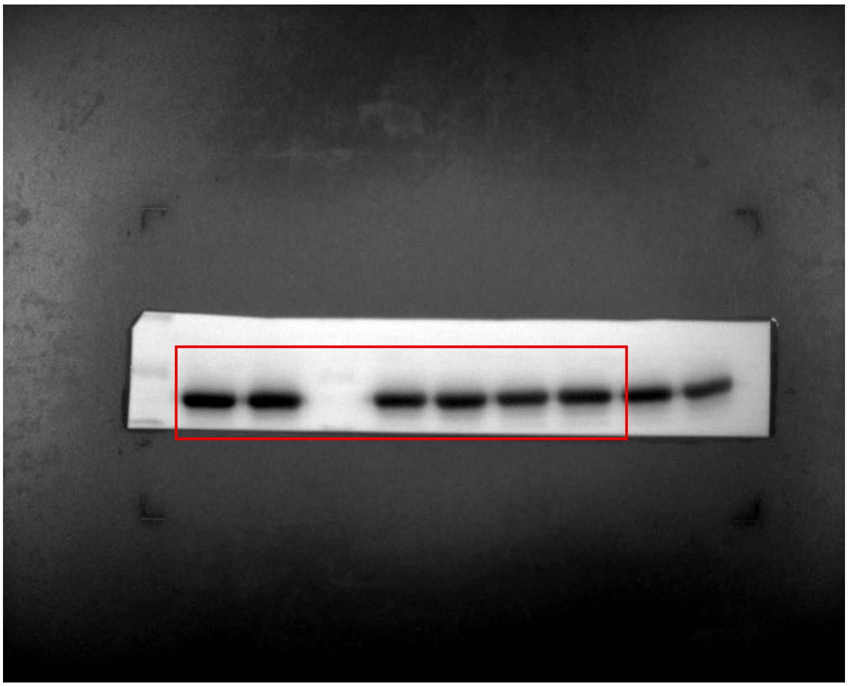

CDK4 (Con, OE)

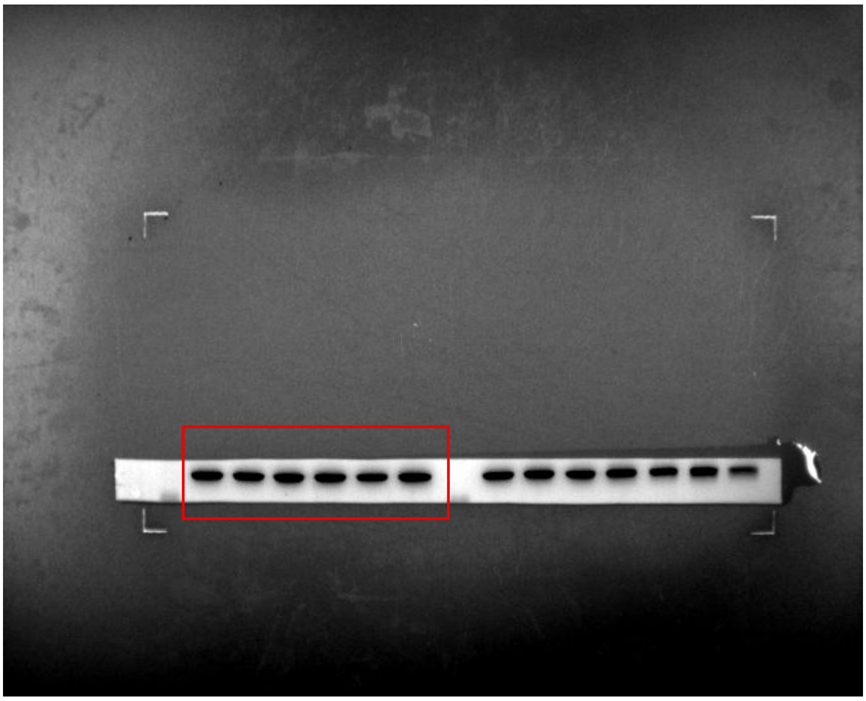

CDK6 (Con, OE)

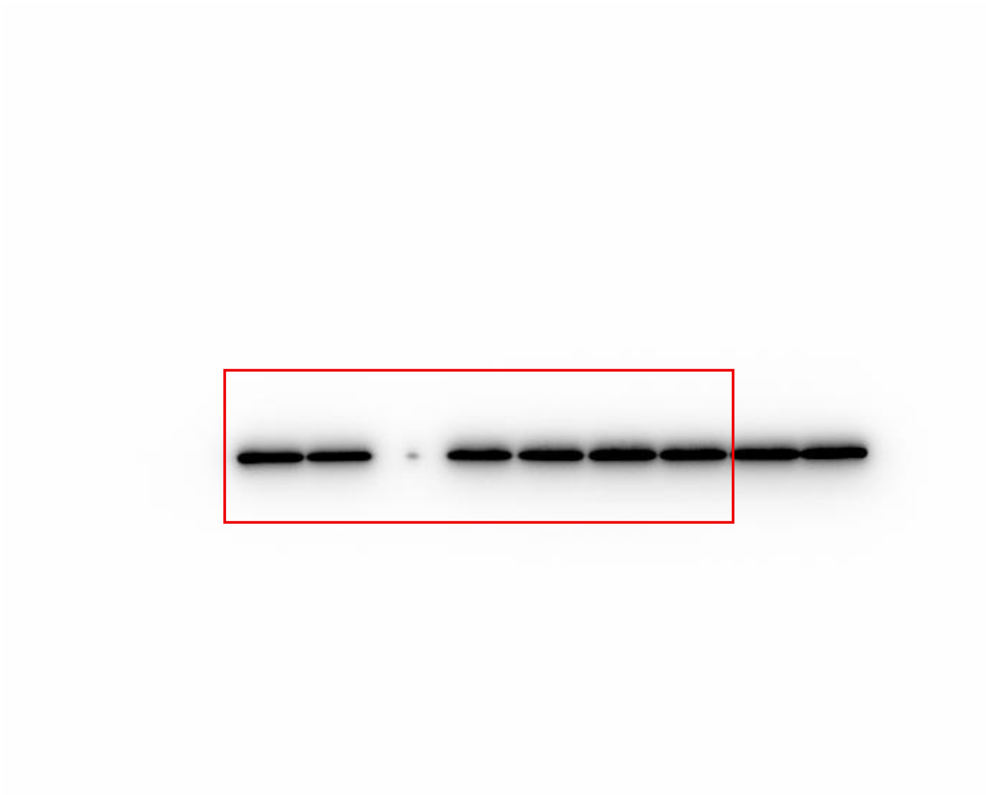

PCNA (Con, OE)

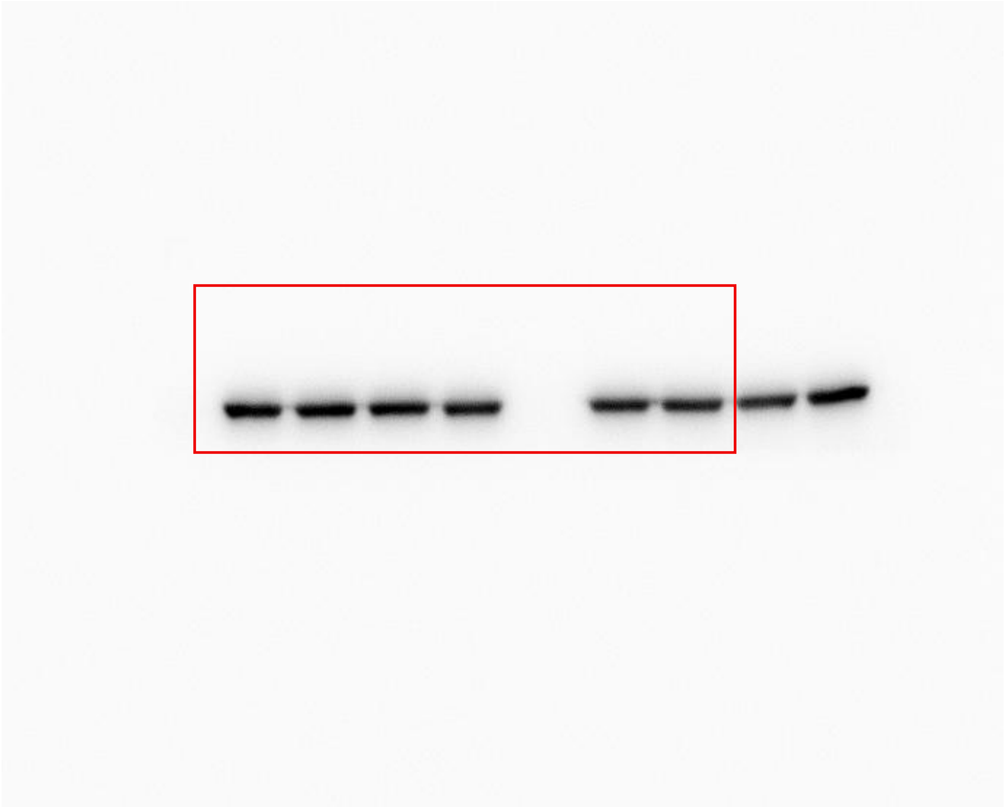

Actin (Con, OE)

G-I

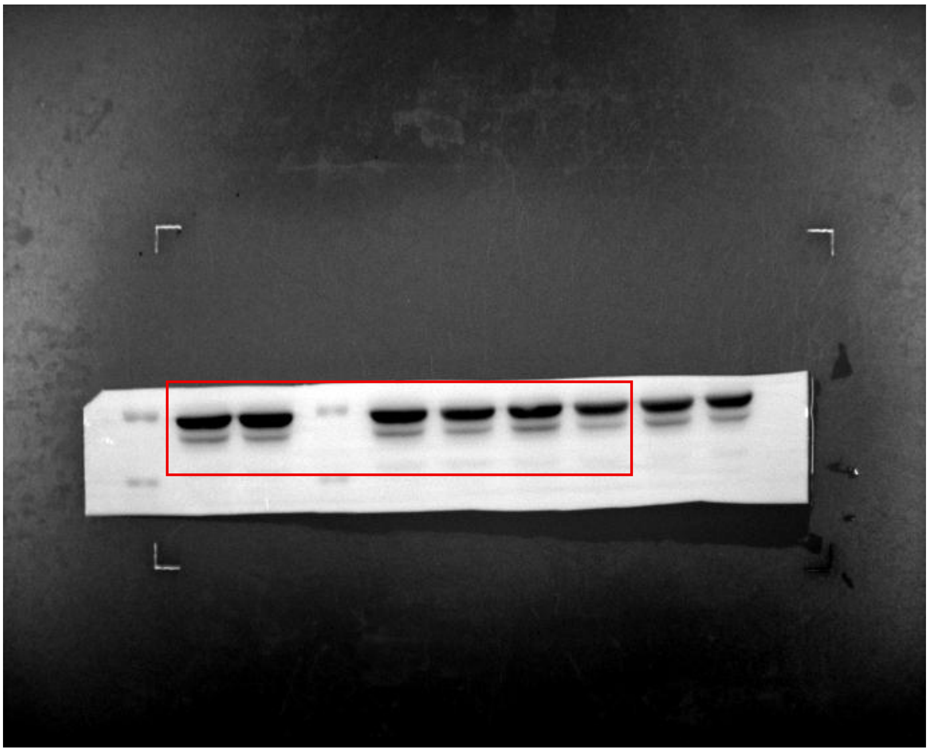

Cyclin A2 (Con, OE)

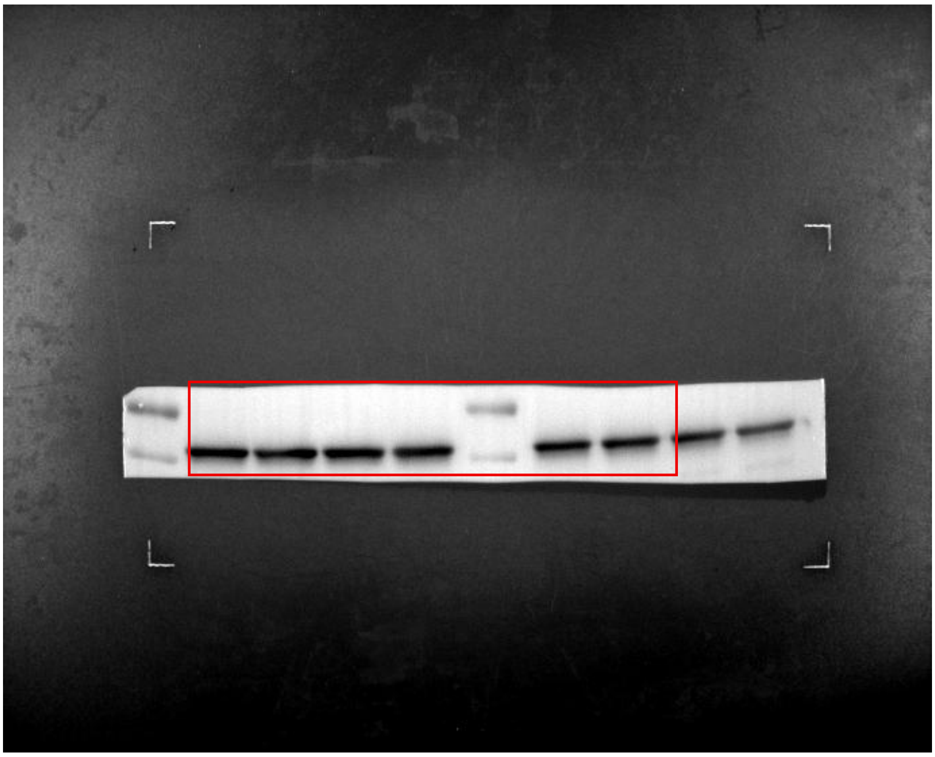

Cyclin B1 (Con, OE)

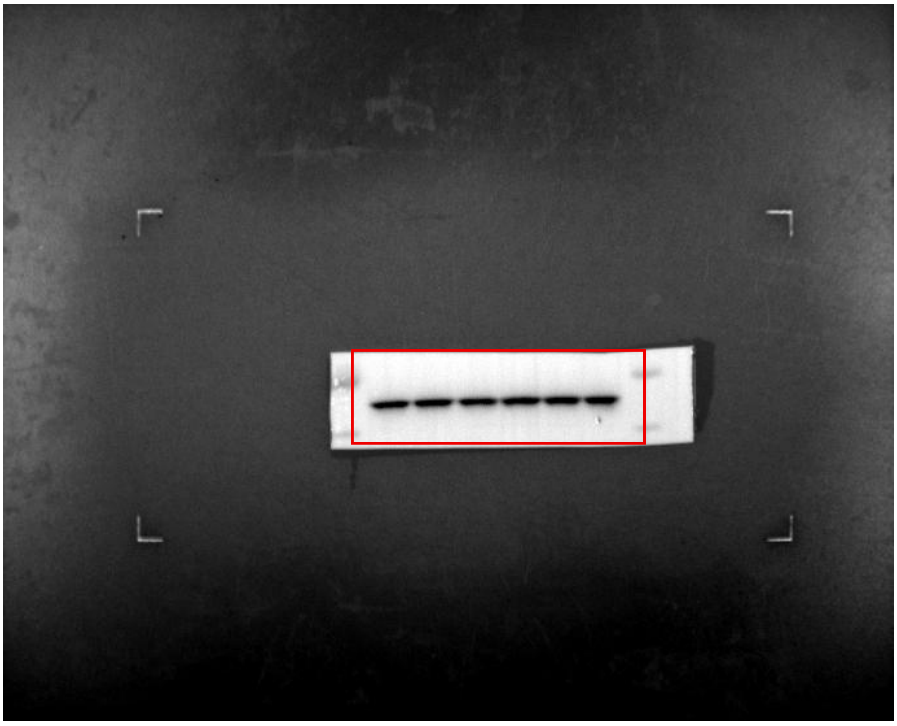

Cyclin B2 (Con, OE)

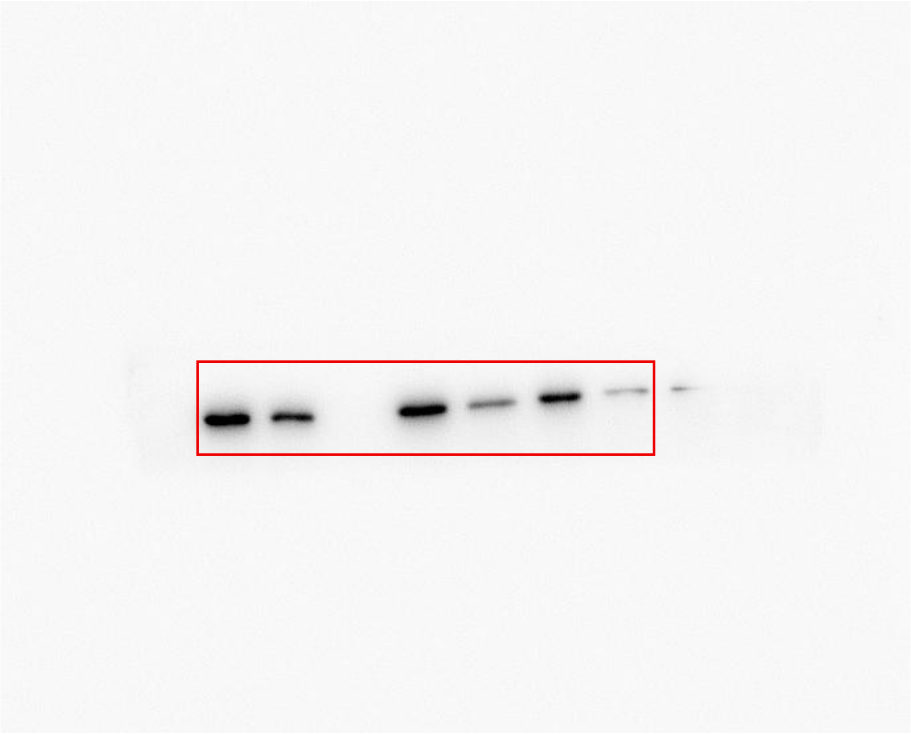

Cyclin D1(Con, OE)

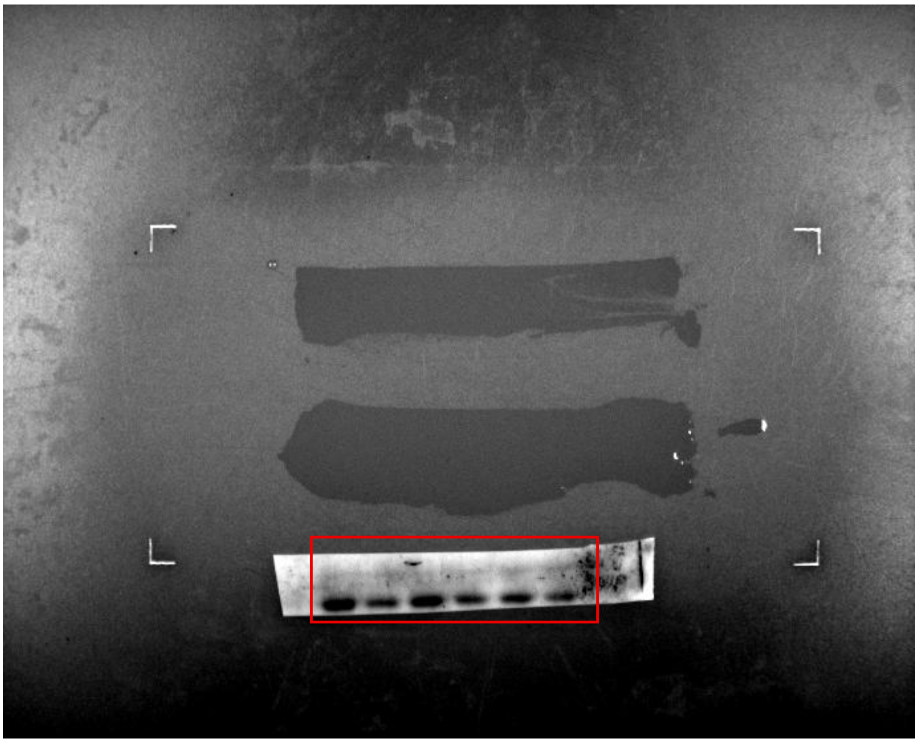

Cyclin D2 (Con, OE)

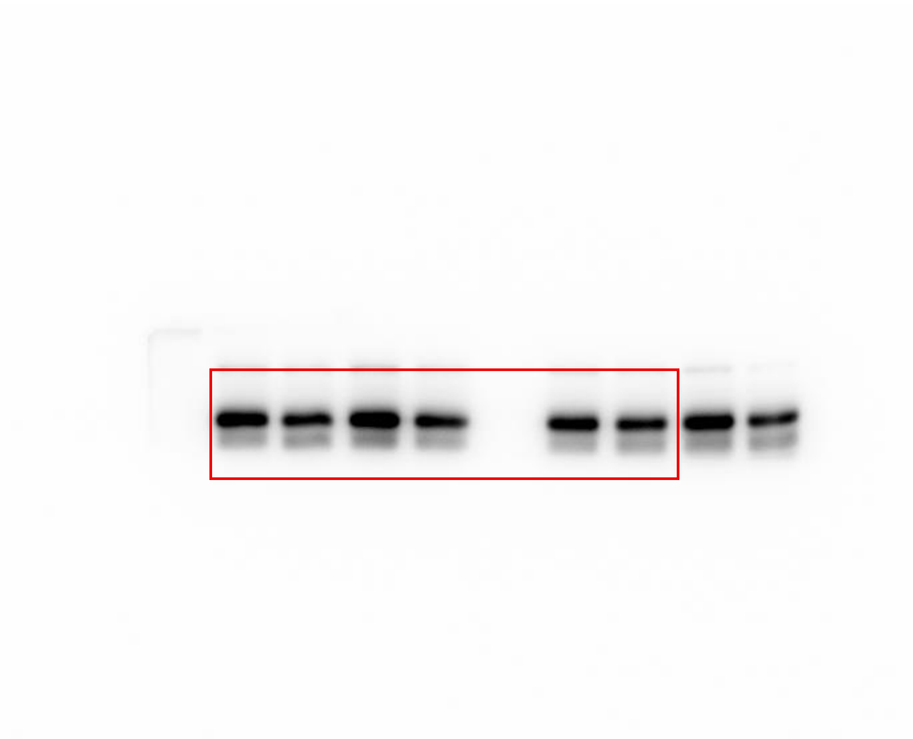

Cyclin E1 (Con, OE)

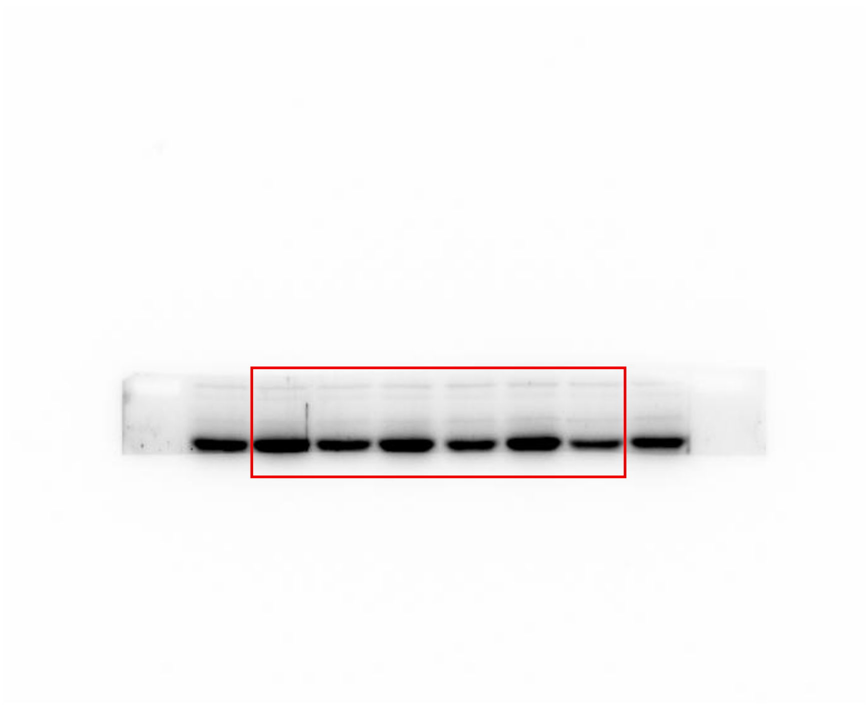

Cyclin E2 (Con, OE)

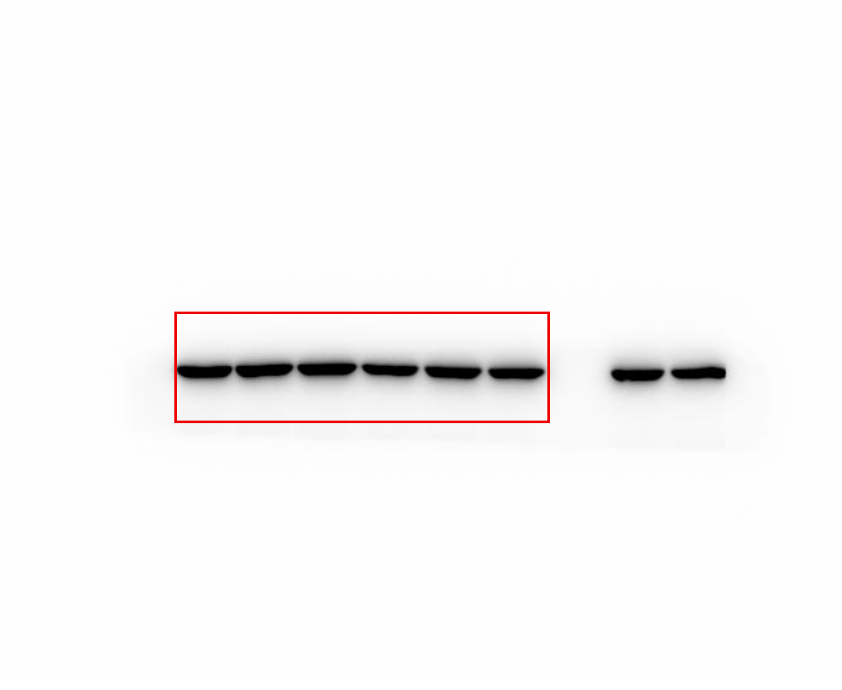

Actin (Con, OE)

Figure S5

A

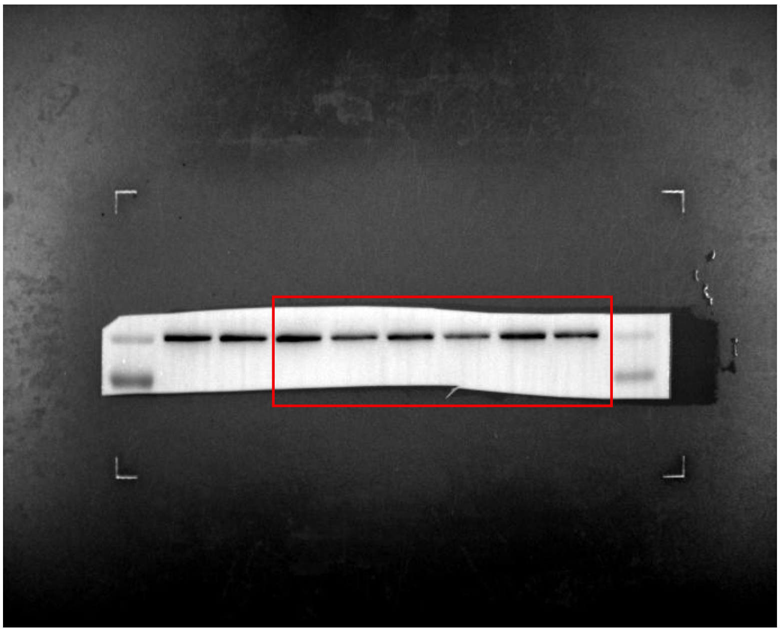

β-Catenin (Con, OE)

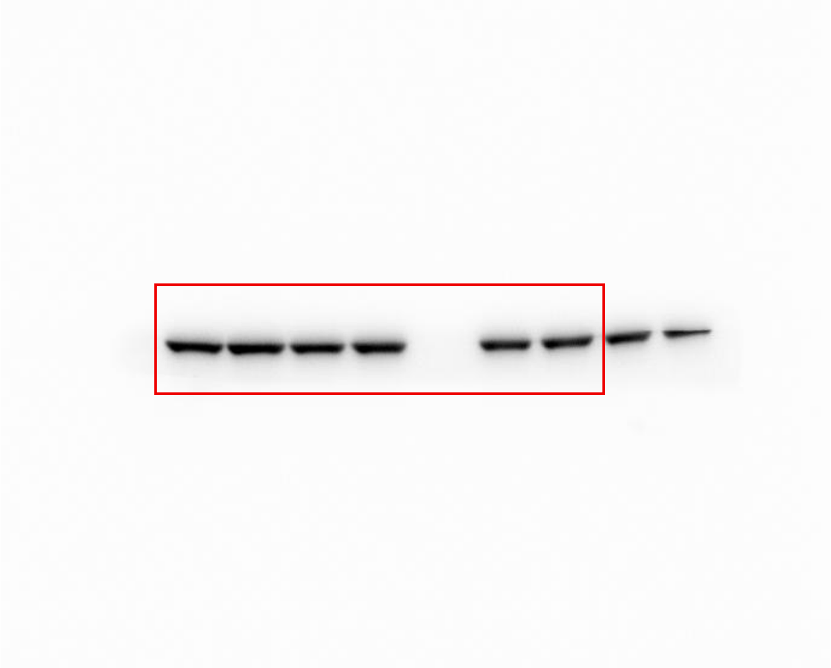

Actin (Con, OE)

E

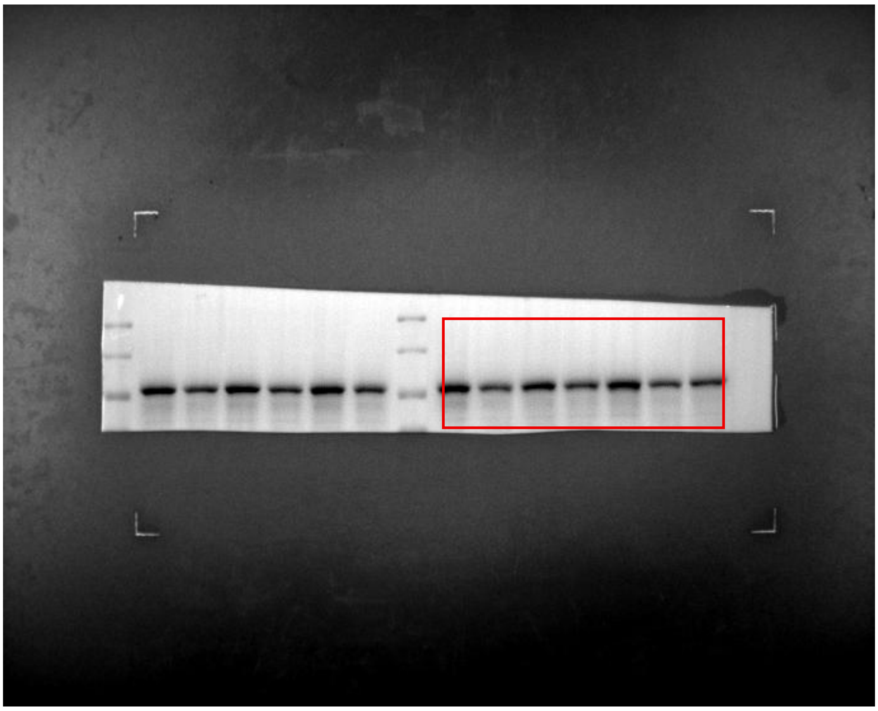

p-β-Catenin (S552) (Con, OE)

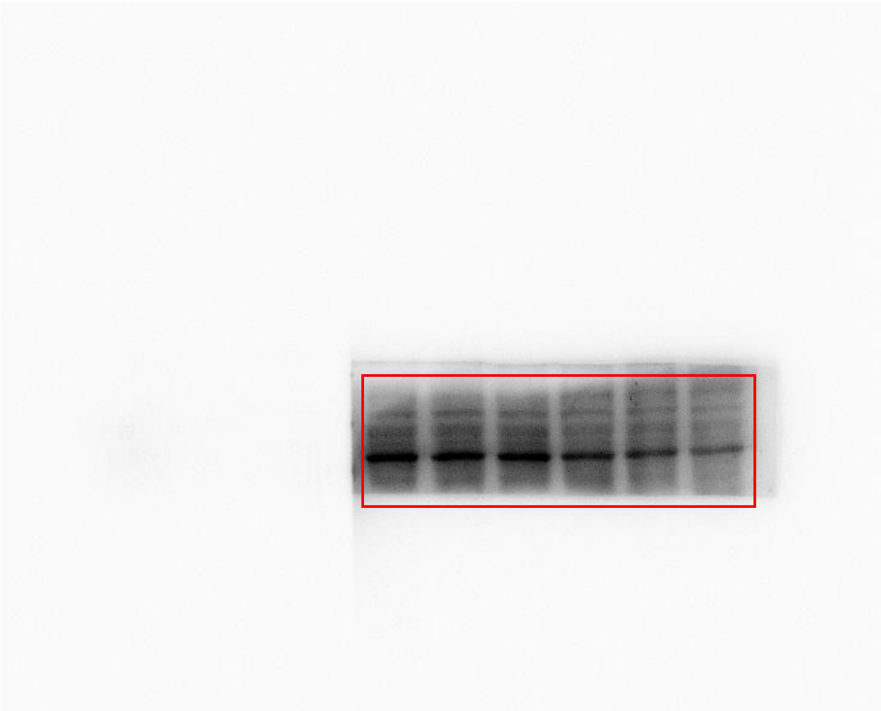

p-β-Catenin (S33/S37/T41) (Con, OE)

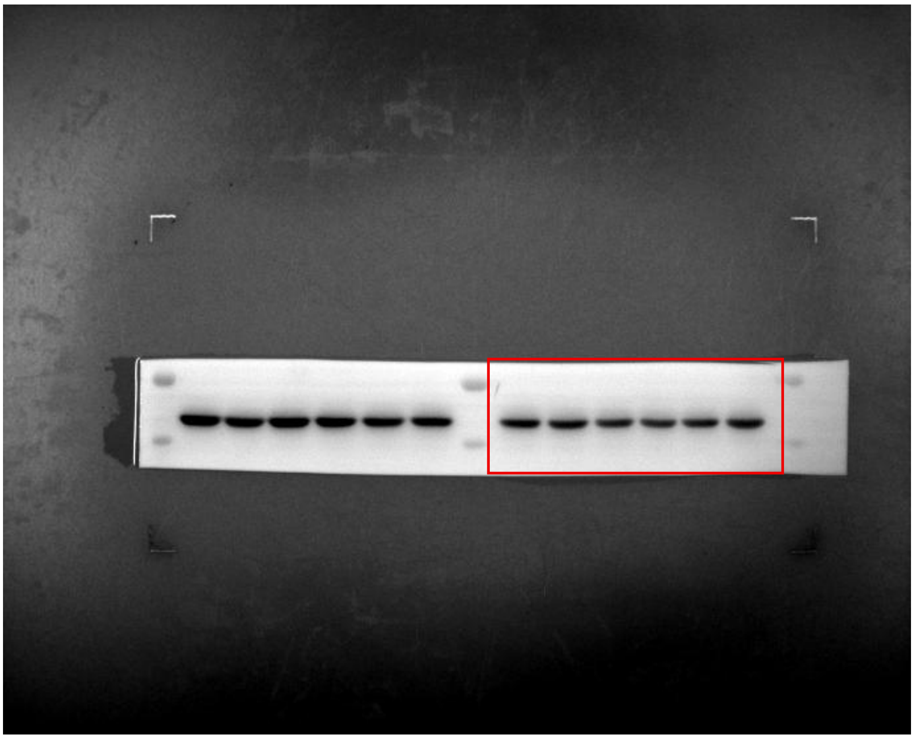

Actin (Con, OE)

Figure S6B

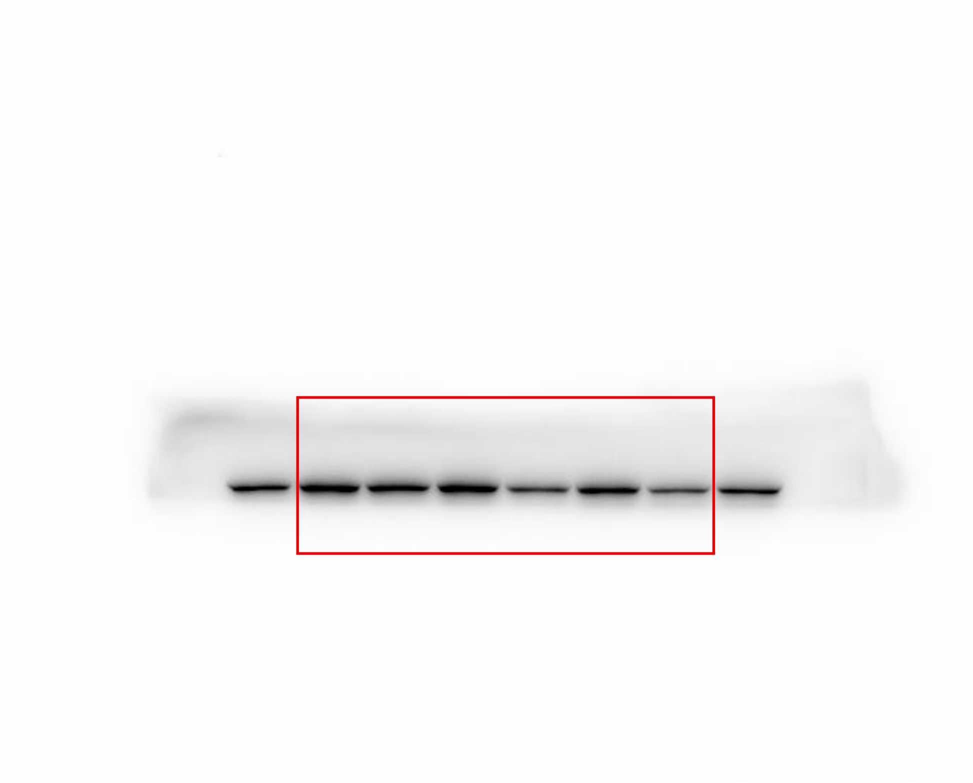

α-Catenin (Con, OE)

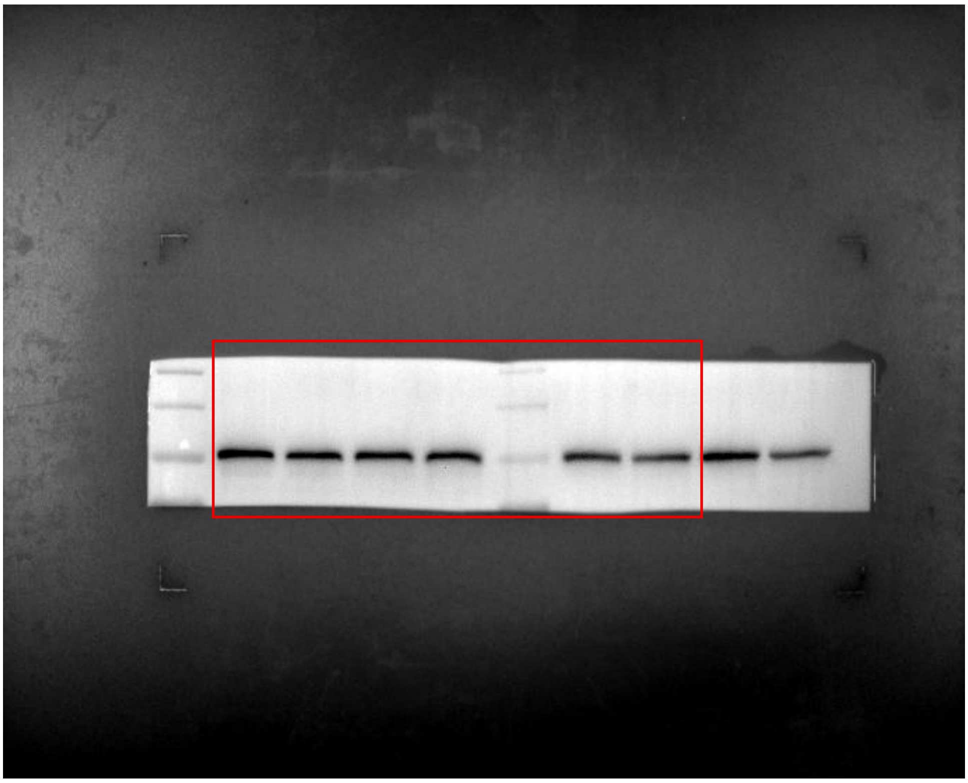

E-cadherin (Con, OE)

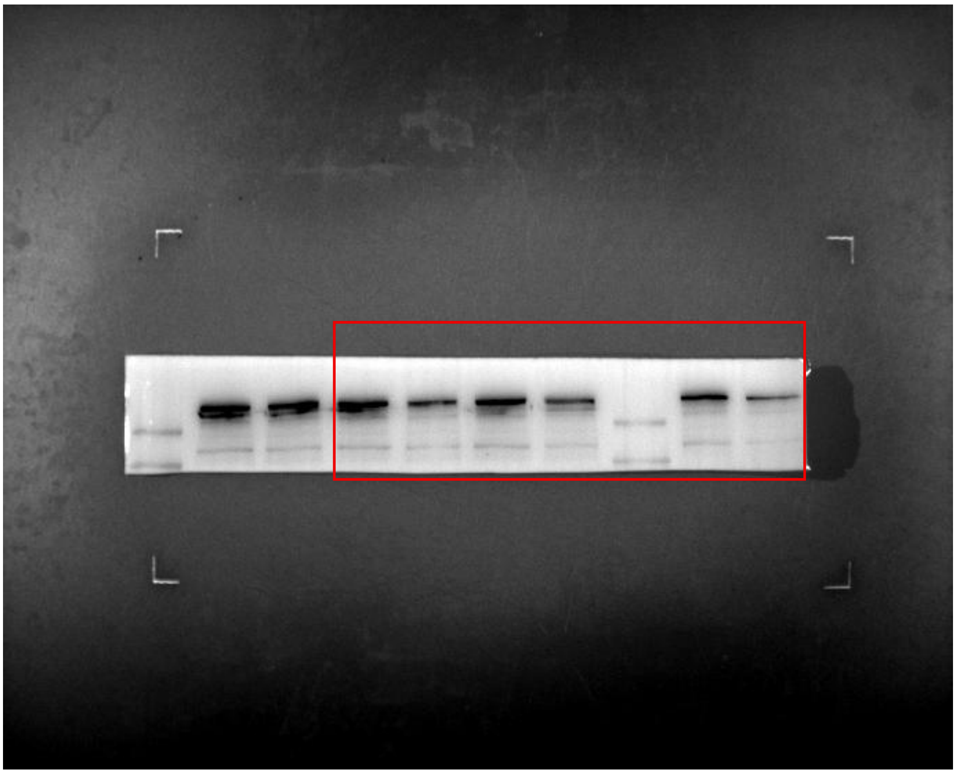

ZO1 (Con, OE)

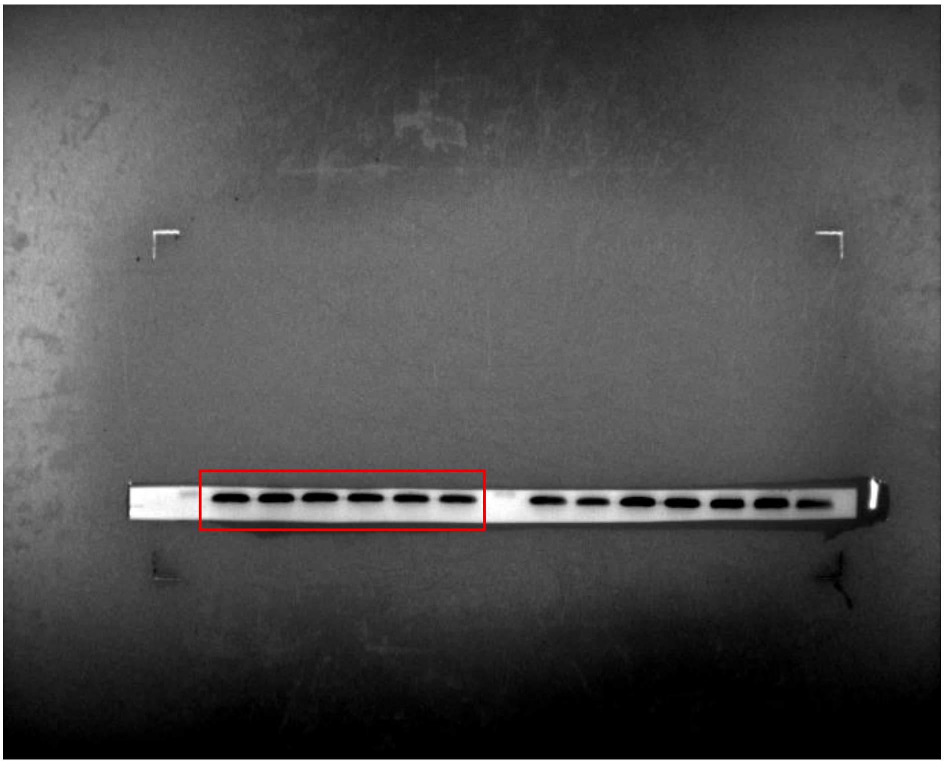

Actin (Con, OE)
