# Supplementary figures and images for: PIWIs Regulate Spermatogonia Self-Renewal and Differentiation by Wnt/β-Catenin Signaling Pathway in Eriocheir sinensis
Source: Biology (Basel). 2025 Oct 18;14(10):1440. doi: 10.3390/biology14101440 (PMC12561773; doi:10.3390/biology14101440)

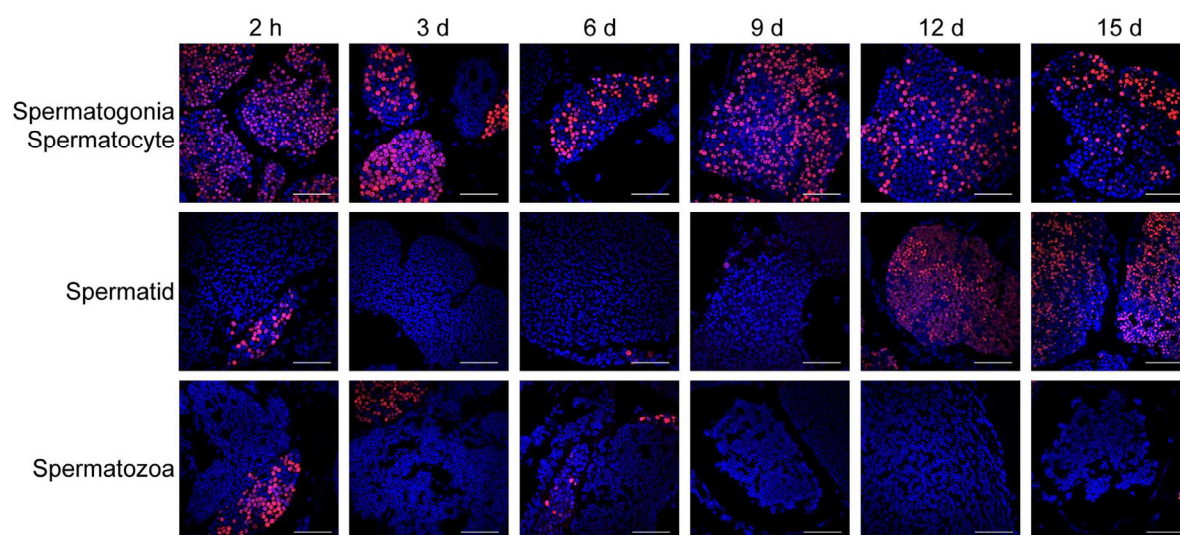

**Figure S1**

Supplement: Supplementary file 1 [file biology-14-01440-s001.zip › supplementary S1.pdf]
